# Five years of patient and public involvement and engagement (PPIE) in the development and evaluation of the Pain-at-Work toolkit to support employees’ self-management of chronic pain at work

**DOI:** 10.1186/s40900-025-00757-5

**Published:** 2025-07-15

**Authors:** Holly Blake, Victoria Abbott-Fleming, Sarah Greaves, Sarah Somerset, Wendy J. Chaplin, Elaine Wainwright, Karen Walker-Bone

**Affiliations:** 1 School of Health Sciences, University of Nottingham, Nottingham, UK; 2 NIHR Nottingham Biomedical Research Centre, Nottingham, UK; 3 Burning Nights CRPS Support, Derbyshire, UK; 4 Faculty of Medicine and Health Sciences, University of Nottingham, Nottingham, UK; 5 School of Medicine, University of Nottingham, Nottingham, UK; 6 Aberdeen Centre for Arthritis and Musculoskeletal Health (Epidemiology Group), School of Medicine, Medical Sciences and Nutrition, University of Aberdeen, Aberdeen, UK; 7 Centre for Pain Research, University of Bath, Bath, UK; 8 Monash Centre for Occupational and Environmental Health, Monash University, Clayton, VIC Australia

**Keywords:** Public involvement, Chronic pain, Work, Digital, Intervention, Self-management.

## Abstract

**Background:**

Patient and public involvement and engagement (PPIE) is essential for the design, delivery and dissemination of high-quality, meaningful research. However, reporting of PPIE contributions is seldom transparent or consistent. We aimed to document and critically reflect on the process of embedding robust PPIE throughout every stage of the research cycle in the co-creation and evaluation of the Pain-at-Work (PAW) Toolkit, a digital resource to support working age adults with self-managing chronic pain at work.

**Methods:**

Using the Guidance for Reporting Involvement of Patients and the Public (GRIPP2-SF) checklist we describe and reflect on PPIE input into four phases of the PAW Toolkit development and testing taking place over five years, all co-led by PPIE-partners, including: (1) Co-Creation: with stakeholder consultation (*n* = 27), surveys with employees (*n* = 274) and employers (*n* = 107), expert peer review (*n* = 40), (2) Prototype Evaluation: with end-user testing (*n* = 104), end-user interviews (*n* = 15), expert peer reviews (*n* = 15), (3) Review and Update: with a public concept mapping exercise (*n* = 20) and expert peer reviews (*n* = 15), (4) Feasibility Testing: with PPIE-partners (*n* = 2), PPIE-members (*n* = 5), PPIE-contributors (*n* = 10).

**Results:**

PPIE was successfully embedded at every stage of the research cycle. Our PPIE-partners co-led activities to gather the views of diverse stakeholders (PPIE-contributors) such as healthcare professionals, employers, and people with lived experience of chronic pain. We outline ‘how’ PPIE took place at each phase, and ‘who’ was involved in each activity. We describe PPIE results in terms of the impact of PPIE on PAW Toolkit development (Phase 1–3) and the research process (Phase 1–4).

**Conclusion:**

Our PPIE partnerships and shared decision-making led to the co-creation, update and evaluation of the PAW Toolkit, an intervention which is appropriate, meaningful and relevant to working-age adults living with chronic pain. We present components for successful PPIE, and map our Pain-at-Work PPIE to recommended components. Components for successful PPIE, challenges and mitigations are reflected upon. PPIE enhanced the ‘real-world’ value of our intervention and methodological rigour of the research processes. Our worked example of PPIE and transferable recommendations could be used to guide other researchers embarking on national or international health research.

**Trial registration (phase 4):**

ClinicalTrials.gov NCT05838677; registered 01/05/2023 https://clinicaltrials.gov/study/NCT05838677.

**International registered report identifier (IRRID):**

DERR1-10.2196/51474.

**Supplementary Information:**

The online version contains supplementary material available at 10.1186/s40900-025-00757-5.

## Abstract (plain language summary)

Patient and public involvement and engagement in research (PPIE) is an essential part of the research process, but PPIE contributions are not always reported in a transparent and consistent way. Here, we present a worked example of how we have embedded PPIE throughout every stage of our research, over a five-year period. Our example is the Pain-at-Work Toolkit, which we designed to support working age adults with self-managing chronic pain at work. The Pain-at-Work Toolkit provides work-related advice and support, such as how to self-manage their pain while at work, and how to access adjustments to their workplace or job role to help them participate in work. We worked together with PPIE-partners to co-create the content and presentation of the Pain-at-Work Toolkit, considering the views of diverse ‘stakeholders’ described as PPIE-contributors (largely healthcare professionals, employers, and people living with chronic pain). This process involved our PPIE-partners co-leading a range of activities, including a public consultation event, two online surveys with people living with pain, and employers, and gathering expert peer reviews of the developed Toolkit. Together with our PPIE-partners, we pilot tested the Toolkit by asking stakeholders and people living with chronic pain to use the Toolkit and share their views in online surveys and interviews. We worked in collaboration with PPIE-partners to use this information to make further updates to the Pain-at-Work Toolkit using a range of methods and approaches. Next, we tested it in a workplace trial, that was co-managed by our PPIE-partner and included 380 people with chronic pain. We have engaged in PPIE throughout the duration of the trial and have two PPIE-partners who are equal members of our research team and co-authors. We have reported our PPIE processes and reflections throughout the five years using the GRIPP2-SF checklist. From what we have learned, we offer practical suggestions for PPIE in future real-world studies.

**Supplementary Information:**

The online version contains supplementary material available at 10.1186/s40900-025-00757-5.

## Background

It is widely accepted that patient and public involvement and engagement (PPIE) is a vital part of health research [1], since it enhances the relevance, appropriateness, quality, and ethical integrity of research [2, 3]. Public involvement can help to identify the most important research priorities, shape study designs to maximise participation, ensure that research is ethically conducted and that research tools are appropriate, increase recruitment and retention rates, and create more accessible information for research participants [3]. There are mutual benefits, since public contributors report increased confidence, a sense of purpose, and feeling valued [4].

Most funding bodies expect that PPIE will be integral to the research processes, and they commonly provide infrastructure, support and guidance [5, 6]. While PPIE is known to benefit research, the reporting of PPIE in research has previously been inconsistent, lacking sufficient detail on the process and impact of PPIE [2, 7, 8]. The availability of PPIE reporting guidelines and checklists, such as the Guidance for Reporting Involvement of Patients and the Public (GRIPP-2) [9], has gone some way to increasing consistency in reporting in recent years. However, the level of detail can be sub-optimal. A mixed-methods analysis of current practice in health research publications found that reporting was commonly incomplete, with only 40% of publications reporting the aim of PPIE, and reports on the influence of patients’ input being “vague” [10]. Poor quality reporting has been observed even when checklists have been adopted [10] and many researchers report only on PPIE benefits without addressing PPIE challenges [11]. Other researchers have identified a complete absence of PPIE reporting. For example, a study of PPIE in clinical trials published in general nursing science journals identified 89 randomised controlled clinical trials published in 2021, in 27 journals, none of which included any statement or evidence of PPIE [12]. Reporting PPIE in research publications has been described as “the exception and not the rule” [13].

There remains a need to enhance the quality of PPIE reporting, providing specific details about *how* and *when* PPIE has been implemented and *by whom*, as well as documenting the *impacts* of PPIE including both benefits and challenges experienced, and how they were managed. This has value for a broad range of stakeholders. Clear reporting ensures that the contributions of public contributors are recognised and valued which could encourage further engagement in research. For researchers, transparent reporting of PPIE not only ensures that studies and interventions are relevant and impactful but may encourage other researchers to engage in high quality PPIE reporting. Higher quality reporting using standardised approaches enhances the credibility and reproducibility of research and therefore creates a stronger evidence base for the influence of PPIE on research processes and outcomes. Improved documentation around PPIE can benefit funders by ensuring that research aligns with public needs. Finally, insights from high-quality PPIE reporting can lead to improved policies and practices, across diverse settings and geographical regions.

In this paper, we provide a worked example of PPIE implementation and reporting, by documenting and critically reflect on the process of PPIE in the context of workplace health research. We show how collaborative working with members of the public, and shared decision-making, led to the co-creation of the PAW Toolkit, and improved the quality, relevance, and appropriateness of the PAW Toolkit and our research processes.

This work highlights the importance of embedding robust PPIE throughout every stage of the research cycle.

### Definitions and terminology

For this study, we use the term ‘patient and public involvement and engagement in research’ and the acronym PPIE. We adopt the INVOLVE definition of PPIE as research which is “carried out ‘with’ or ‘by’ members of the public rather than ‘to’, ‘about’ or ‘for’ them” [14]. Our PPIE occurs at every stage of the research process, from identifying the research question through to influencing policy makers by dissemination of results. We refer to ‘PPIE-partners’, a mutually agreed term which demonstrates an established and equal partnership with these members of our research team. Having a PPIE-partner confirms our commitment to a collaborative approach in our applied research. Our research programme has included two ‘PPIE-partners’ (SG, VAF) who have contributed at different stages of our research programme and are considered equal members of the research team. They were recruited through the research team’s professional networks. Our PPIE-partners are members of the public with lived experience of chronic conditions and knowledge of the impact of chronic conditions (including chronic pain) on work. One PPIE-partner has held this position throughout Phases 1–4 (VAF), the other through Phases 1–3 (SG). We have ‘PPIE-members’, who hold positions on our study advisory or steering groups. Our PPIE-partner involved in Phase 1–3 activity transitioned to become PPIE-member in Phase 4. Those supporting involvement or engagement for specific phases or steps within the presented activity (but are not PPIE-partners or PPIE-members) are referred to as ‘PPIE contributors’ or ‘public contributors’. By ‘involvement’ we refer to research carried out ‘with’ or ‘by’ people with lived experience of chronic pain or relevant stakeholders, such as representatives of employing organisations or professional bodies. By ‘engagement’ we refer to awareness raising, sharing, disseminating knowledge about research, and engaging people with lived experience of chronic pain (not necessarily patients), and other members of the public in a conversation about research.

### Research context

The research which is the context for this PPIE activity relates to the development and evaluation of a digital intervention called the Pain-at-Work (PAW) Toolkit [15, 16]. The PAW Toolkit aims to support people who have ‘chronic or persistent pain’ in their place of work (referred to as ‘chronic pain’ hereon). The rationale for focusing in this area is that existing self-management tools for people with chronic pain tend to focus on symptom reporting, treatment programmes or exercise and do not address barriers to work, facilitators of work ability, or workplace pain self-management strategies. The PAW Toolkit addresses this intervention and support gap. The idea for the PAW Toolkit came from a discussion between the lead author and five working adults who experience chronic pain. These individuals reported back pain, shoulder pain, knee pain, fibromyalgia, and migraine, and worked in the public or private sector, three in large organisations and two in small-to-medium sized enterprises (SMEs). All felt that there was not enough support in the workplace for people with chronic pain, to help them manage their condition and enjoy a good quality working life. We discussed possible solutions such as occupational health services and employee workshops. The former was proposed as one route to providing support, but provision is inequitable as occupational health services are not available in all employment settings. The latter was seen to be potentially informative, but employee workshops are time and resource intensive. There was consensus that a digital toolkit would be the most flexible, accessible and low resource approach to providing support in different types of workplaces, although a systematic review showed that there were no evidence-based digital resources available at that time [17].

**Fig. 1:**
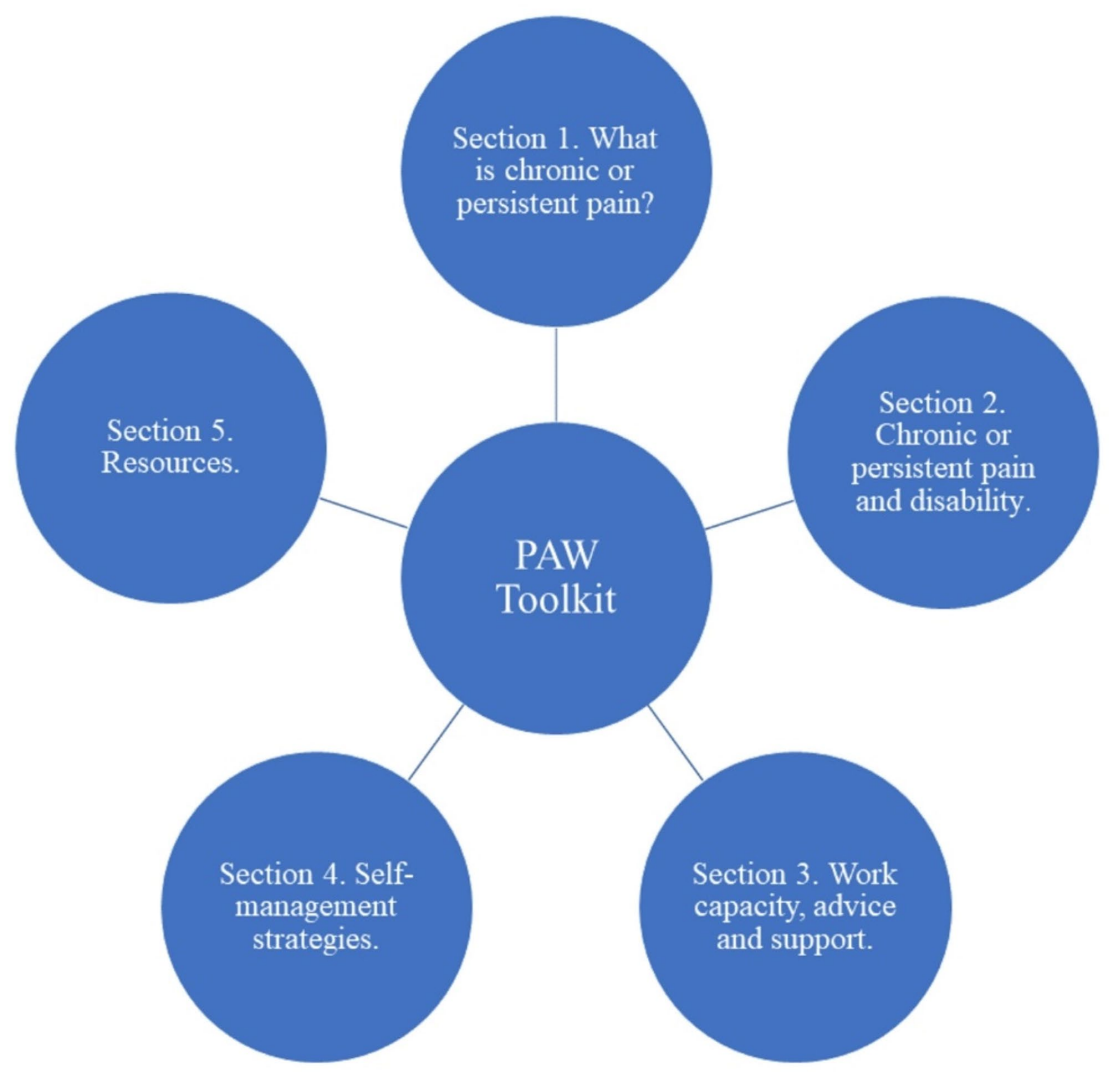
Pain-at-Work Toolkit sections. (Source: [15])

The PAW Toolkit is fully described elsewhere [15, 16]. It is designed to be relevant to any employee with chronic pain in any organisation type, size, or sector. The PAW Toolkit offers evidence-based advice on chronic pain, disability rights, work capacity, pain self-management strategies, and signposting to support (Fig. 1). The theory of change for the PAW Toolkit is: “Providing employees with access to the PAW Toolkit will increase knowledge about employee rights, how to access support for managing a painful chronic condition in the workplace, and lifestyle behaviours that facilitate the management of chronic pain. This in turn will lead to improved self-management of pain at work. The ultimate aim is to improve outcomes for individuals (self-efficacy, work ability, job perceptions, health, and wellbeing) and organisations (presenteeism, absenteeism)” [15, 16].

## Methodology

Here, we include an overview of all PPIE stages during the development, evaluation and feasibility testing elements of our research programme, describing and reflecting on partnership and shared decision-making with members of the public. Reporting is structured using international evidence based, consensus informed guidance for reporting patient and public involvement in research called GRIPP2-SF [9]; Guidance for Reporting Involvement of Patients and the Public, Version 2, Short-form. The GRIPP2-SF (Additional file 1) aims to improve quality, transparency, and consistency in PPIE reporting and includes five items on (i) aim, (ii) methods, (iii) results, (iv) discussion and conclusions, and (v) reflections/critical perspective.

### Aim of the PPIE

In this paper, we aimed to use internationally recognised evidence-based guidance to document and critically reflect on the process of embedding robust PPIE throughout every stage of the research cycle in the co-creation and evaluation of the PAW Toolkit, a digital resource to support working age adults with self-managing chronic pain at work. The purpose of the PPIE input into our research programme was to co-create the PAW Toolkit, and to improve the quality, relevance, and appropriateness of the PAW Toolkit and our research processes to our target population - working age adults with chronic pain.

We aimed to reflect on components for successful PPIE, challenges and mitigations, and provide a worked example of PPIE and transferable recommendations that can be used to guide other researchers embarking on national or international health research.

### Methods of the PPIE

**Fig. 2:**
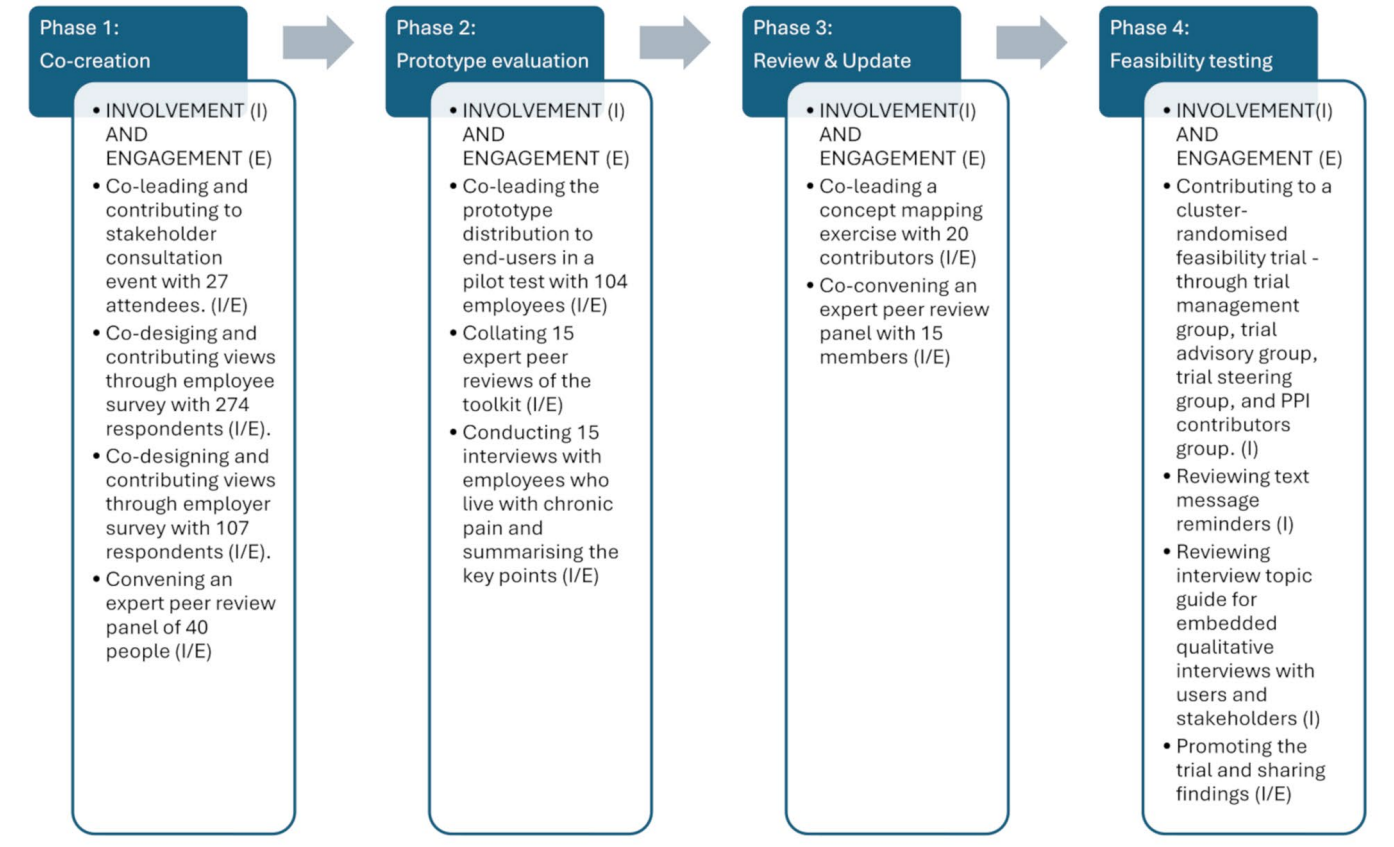
Four phases of PPIE

The four phases of the PAW Toolkit development and testing included: (1) Co-creation, (2) Prototype Evaluation, (3) Review and Update, and (4) Feasibility Testing. Our PPIE activity occurred across all four phases - Fig. 2 identifies the public involvement and/or engagement at each stage, identifying where members of the public have co-designed, co-led and contributed to activities within each phase. Further details of the research context, including the intervention’s theoretical underpinning, content, and presentation, and all processes involved in the co-creation, update and testing of the PAW Toolkit, are reported elsewhere [15, 16, 18]. For each phase we describe the purpose, who was involved and the approaches taken.

### Phase 1 co-creation

The purpose of Phase 1 was for the researchers to work in partnership with members of the public to co-create the PAW Toolkit content, technical presentation and delivery approach. We used an agile approach to digital intervention development, which the lead author has used previously in the development of digital interventions in the context of work and health [19, 20, 21]. Phase 1 involved 4 steps of PPIE activity [16], and five co-authors were involved in each step within this phase (HB, SS, WJC, SG, VAF). In Step 1, a PPIE-partner co-led a stakeholder consultation workshop with 27 attendees. In Step 2, PPIE-partners and the researchers co-designed a stakeholder survey through which 274 working adults (‘employees’) with chronic pain (PPIE-contributors) shared their views. In Step 3, PPIE-partners and the researchers co-designed a second stakeholder survey through which 107 employer representatives from 45 small-to-medium sized organisations and 62 large organisations (PPIE-contributors) shared their views. In Step 4, a PPIE-partner convened a peer review panel of 40 stakeholders.

**Fig. 3:**
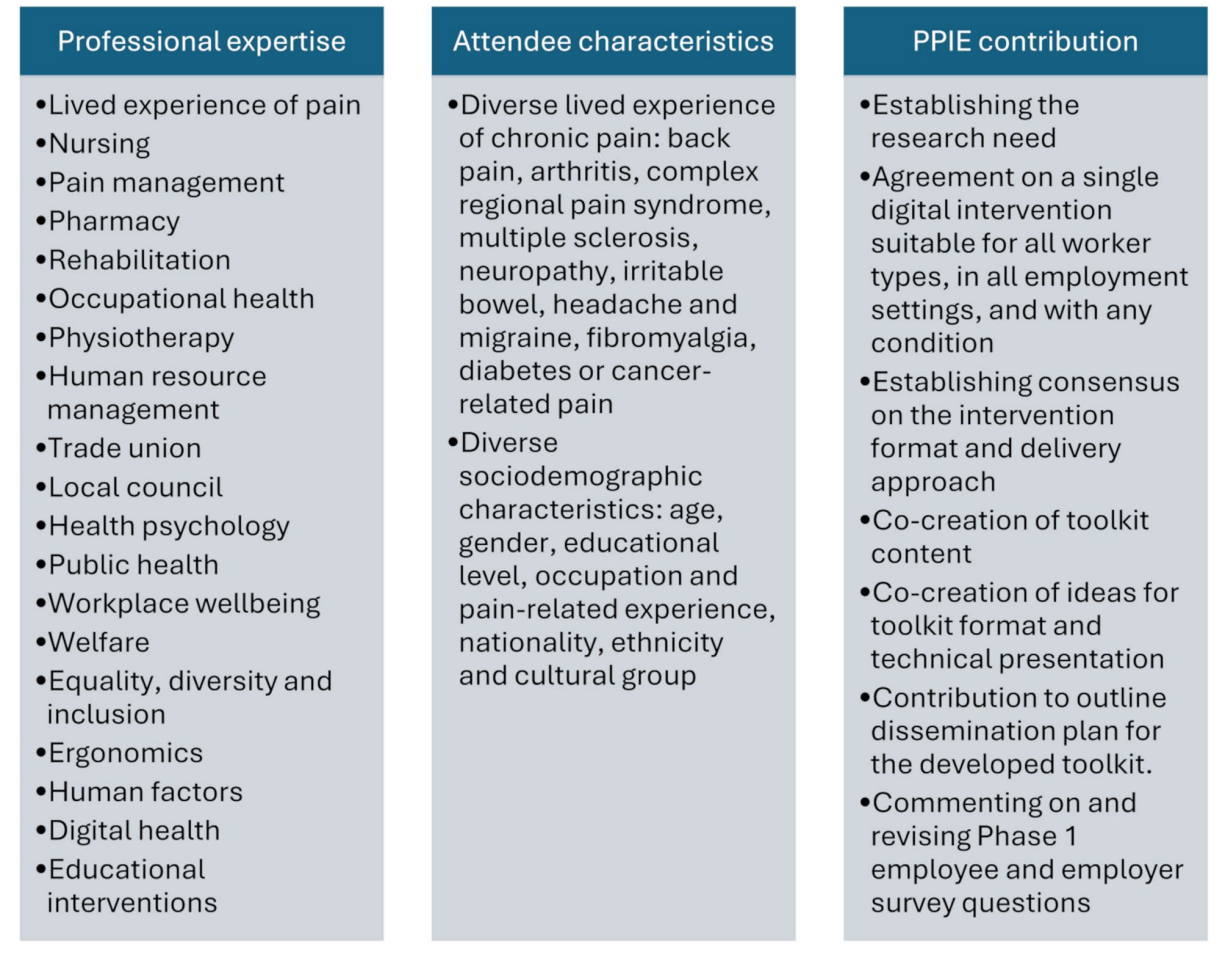
Phase 1 PPIE consultation: stakeholder backgrounds and contributions

Step 1, the stakeholder consultation workshop was a 2-hour face-to-face event, held on 04 February 2020 in a room at a higher education institution, in England. Stakeholder consultation workshops are common method for gathering input and feedback from members of the public for intervention co-production, as part of a wider approach to PPIE [22]. The purpose of the event was for ‘stakeholders’ (e.g., health professionals, employers and members of the public with lived experienced of chronic pain) to share their views about the type of content they would value, and what format they would prefer it in The event involved a presentation about the topic area (chronic pain self-management) and the concept (a digital toolkit) delivered by the lead author (HB). This was followed by discussion and small group activities, facilitated by the project researchers (HB/SS – full room discussion; WJC – group activities), and PPIE-partner (SG – group activities). In total, twenty-seven public contributors attended the consultation. Their characteristics, expertise and PPIE contributions are shown in Fig. 3, which highlights areas of shared decision-making. At attendees’ request, these demographics are documented at group level to protect the confidentiality of those public contributors who had disclosed chronic pain conditions.

The remainder of PPIE activity for Phase 1 was conducted during the coronavirus (COVID-19) pandemic which occurred between 11 March 2020 and 05 May 2023. As such, all other activities were conducted remotely, and the project timelines were extended.

In steps 2 and 3, two PPIE contributors (VAF/SG), co-designed and undertook two online surveys together with members of the research team (HB/SS) to gather views and suggestions from working adults with chronic pain (step 2), and employer representatives from different types and sizes of organisation (step 3). According to the National Institute for Health and Care Research Applied Research Collaboration East Midlands [22], surveys are a commonly used and time-efficient PPIE advisory method, to gather opinions and perspectives to shape research – in this case, toolkit development. Potential public contributors could access the questions through a link to an online survey which was circulated on social media, and via professional networks (by HB, SS, VAF, SG).

Employees were asked to share their support needs and suggest how employers might meet these needs; employers were asked about the best ways to support people with chronic pain at work and to share resources and materials that could be included in a toolkit.

Views were gathered between 02 March – 30 April 2020; due to the pandemic, the employee survey was then re-opened for a further three months between 01 October 2020–31 December 2020 to provide opportunity to gather further views. As public contributors, a total of 274 employees, and 107 employer representatives shared their views. Full details are reported in [16]. Suggestions were pooled and summarised by team members working in partnership (researchers: HB, SS; PPIE-partners: SG, VAF) to inform toolkit content development.

Step 4, the expert peer review for Phase 1, was undertaken between 01 October 2020 and 30 November 2020 (iteratively, as per Agile approach). In this step, PPIE-partners (SG, VAF) and researchers (HB, SS) convened an expert peer review panel of 40 stakeholders. These individuals were identified by the research team with input from PPIE-partners. They were chosen for their expertise in chronic pain and disability, work and health, or digital health interventions. They included healthcare professionals, employer representatives, and people with lived experienced of chronic pain. These 40 public contributors reviewed the draft toolkit content and made suggestions for revision, materials to include, and presentation to ensure the toolkit met accessibility guidelines. Further details about Phase 1, including specific detail about changes made to the toolkit during this time, are reported elsewhere [16].

Overall, Phase 1 resulted in a co-created prototype toolkit by 31 December 2020, ready for testing and evaluating. All processes in Phase 1 involved working in direct partnership with our PPIE-partners, who engaged in shared decision-making with the research team and helped to gather the views of diverse stakeholders to inform intervention development.

### Phase 2 prototype evaluation

The purpose of this phase was to evaluate the prototype toolkit. This activity took place between 01 September 2021 and 31 December 2021. Three co-authors were involved in Phase 2, including one researcher (HB) and two PPIE-partners (SG, VAF). The process involved prototype distribution, collating feedback through an online evaluation survey, qualitative interviews and expert peer review. Surveys and interviews are described as commonly used PPIE advisory methods in the UK [22]. The process was co-led by our PPIE-partner (SG) who gathered and summarised evaluation feedback and conducted the evaluation interviews with supervision and training from the lead author (HB).

Over a 12-week period, the team (researcher: HB; PPIE-partners: VAF, SG) released the web link to the prototype toolkit together with a link to an online evaluation feedback form (Additional file 2). To gather feedback from as diverse a group of public contributors as possible, these links were made accessible to people with lived experience of chronic pain and professionals with expertise in pain management or work and health (‘expert peer reviewers’). Public contributors were able to get involved through information shared via national charities, professional networks, community group newsletters, and social media (*X* (formerly Twitter), Facebook, LinkedIn). Fifteen professionals agreed to be ‘expert peer reviewers’ and share their views – they had expertise in human resource management, occupational health, physiotherapy, nursing, and pain research. One hundred and four working adults who self-identified as living with chronic pain shared their views. To do this, all completed the online feedback form. They were 84% female, 11% male, 5% non-binary and identified with 11 different ethnic groups (Additional file 3).

Thirty of the 104 individuals with lived experience of chronic pain agreed to take part in an interview (with PPIE-partner SG). Of these, 15 were able to find a convenient date and time to do this between 01 October 2021–31 December 2021. The primary purpose of the interviews was to provide a space for public contributors to share more in-depth views on the relevance, usability and utility of the PAW Toolkit. However, this also provided an opportunity for our PPIE-partner (SG) to gain professional experience of leading a PPIE activity.

Interview discussions ranged from 18 to 41 min (mean: 31 min). The 15 public contributors with lived experience of chronic pain included 12 women and 3 men, identifying as White/British (*n* = 14) and White/European (*n* = 1). They were aged between 18 and 64 years (18–24: *n* = 1; 25–34: *n* = 4; 35–44: *n* = 1; 45–54: *n* = 7; 55–64: *n* = 2). The number of years they had experienced chronic pain varied from 2 to 35 years (2–3 years: *n* = 3; 4–6 years: *n* = 5; 8–15 years: *n* = 3; 21–35 years: *n* = 4). One of the public contributors was on long-term sick leave, six were working full-time, and seven were working part-time. These individuals worked in the private sector (*n* = 5), public sector (*n* = 8) and third sector (*n* = 1). Two were self-employed, two were employed in medium-sized organisations (50–250 staff) and 10 were employed in large organisations (> 250 staff).

Our PPIE-partner (SG) summarised the key points within the interview discussions. These were finalised through discussion between two team members (researcher: HB; PPIE-partner: SG), and direct quotes from public contributors were extracted to support and illustrate key points.

Overall, Phase 2 evaluation surveys (from people living with chronic pain, and expert peer reviewers) and interviews (with people living with chronic pain) provided key insights from public contributors which informed updates to the prototype PAW Toolkit. All processes in Phase 2 involved working in direct partnership with our PPIE-partner, who engaged in shared decision-making with the research team and helped to gather the views of diverse stakeholders to refine the PAW Toolkit prototype.

### Phase 3 review and update

The COVID-19 pandemic led to a delay between PAW Toolkit completion [16] and its testing in a feasibility trial [15]. The toolkit was developed in 2020 and evaluated in 2021 (Phases 1 and 2) and we therefore added additional PPIE activity to ensure the toolkit remained fit-for-purpose post-pandemic. The purpose of Phase 3 was therefore to ensure that content remained relevant and up to date, and undertake any updates required (Phase 3).

Phase 3 PPIE was conducted in two steps [18]: (1) A concept mapping exercise involving 20 public contributors occurring in 2022, (2) Expert peer review involving five public contributors occurring in 2022 and 2023.

In step one, a rapid and pragmatic group concept mapping process was undertaken on 14 July 2022, to integrate perspectives of a range of stakeholders with differing experiences and expertise to re-affirm content priorities, update and refine the PAW Toolkit ready for testing in a feasibility trial. This step was co-led by our PPIE-partners (VAF, SG) working together with researchers (HB, SS). Concept mapping is a structured conceptualisation process involving multiple stages: preparation, brainstorming, sorting, rating, and interpretation. It is a commonly used tool for public health intervention development, allowing for the integration of practical and scientific knowledge with stakeholders representing different perspectives, the documentation of programme elements and how they relate to one another, and the identification of priorities [18, 23, 24]. This activity was hosted online in a scheduled slot within an educational event on “Work and Health” being delivered by the lead author (HB). The 20 attendees were public contributors (‘stakeholders’) who were employees with chronic pain, managers, trade union representatives, human resource and occupational health specialists, university researchers and healthcare professionals. All these public contributors were involved in review of the theory of change, generation of statements, sorting, and rating. Six of the public contributors were subsequently involved in mapping and ‘interpretation’ at a follow-up online validation meeting, one week later.

‘Preparation’ occurred prior to the session (between 07 July 2022 and 13 July 2022) and involved the 20 public contributors reviewing the PAW Toolkit accessed via a web link, and theory of change statement (see ‘Research context’); both of which were perceived to be appropriate, relevant and useful. ‘Brainstorming’ took place during the first online session. It involved using free-text responses from the 274 employee survey responses gathered in Phase 1 [16] from which the group generated 102 statements indicating the education and support priorities of people with pain with specific relation to the workplace. After removal of repetition, 78 statements remained. During ‘Sorting’ and ‘Rating’, the 20 public contributors individually sorted statements into meaningful groupings and rated them in terms of perceived importance, and confidence that a web-based toolkit could address each (using 5-item Likert scales: ‘importance’ and ‘confidence’). Categorisation of the statements demonstrated that they all directly aligned with five ‘clusters’ which constituted the five sections of the PAW Toolkit shown in Fig. 1. Insights gathered from this group concept mapping exercise, co-led with PPIE-partners, were used by the research team to support the refinement and updating of the PAW Toolkit. Core sections from the original prototype aligned with the concept mapping outcome and were retained, while section content was updated to reflect current views of members of the public and other stakeholders. Full details of this approach have been published elsewhere [18].

In step 2, the research team gathered feedback from five expert peer reviewers between 10 December 2022 and 15 March 2023, to ensure the content remained relevant in 2023. This process was co-led by our PPIE-partner (VAF) and the lead author (HB) who identified appropriate reviewers and helped to collate feedback. The peer reviewers included three people who lived with chronic pain, and two people with professional expertise in workplace health and wellbeing, specifically, support for people with chronic pain (occupational health specialist, workplace physiotherapist). This process involved online discussion via video-conferencing platform and/or feedback provided via email correspondence.

In summary, Phase 3 PPIE [18] confirmed the validity of the original theory of change and the appropriateness of the PAW Toolkit sections and content. These vital public contributions led to minor revisions to the PAW Toolkit to correct any broken links and add some new information to the additional resources section. At the end of Phase 3, the PAW Toolkit was considered ready for testing in a feasibility trial. Through co-leadership of Phase 3 activities, we demonstrate how our PPIE-partners were involved in shared decision-making relating to the toolkit development and refinement.

### Phase 4 feasibility testing

The purpose of this phase is to ensure that members of the public and relevant stakeholders contribute to the design, processes and dissemination within an externally funded cluster-randomised workplace trial [15]. The aim of the trial was to examine the feasibility and acceptability of the PAW Toolkit with employees who live with chronic pain, in different types of employment settings. The trial opened on 01 March 2023, and recruitment is now complete. From 18 organisations taking part in the trial, 380 employees with chronic pain were recruited. PPIE is embedded at every stage of the research from development, to testing in a trial, dissemination, and informing future research. Seven co-authors have been involved in the PPIE in Stage 4. These individuals are researchers who have planned and coordinated PPIE input to the project (HB, EW, WJC, KWB), a PPIE-partner (VAF) who has led trial-related PPIE activity, and the remaining two co-authors have contributed in an external advisory capacity alongside people with lived experience of chronic pain, through the Trial Advisory Group (TAG) (PPIE-partner: SG) and Trial Steering Group (TSG) (researcher: SS). Our PPIE-partner (VAF) sits on our Trial Management Group (TMG) and provides advice to the study team as an equal partner. During the trial design stage, members of the research team (HB and EW) consulted with 10 PPIE-contributors from across two Versus Arthritis Pain Centres in the UK who reviewed our study materials at the outset (e.g., participant information sheets, protocol, recruitment plans, study summaries) and shared their views on best approaches to recruit and retain organisations and employees. Five PPIE-contributors reviewed additional study materials (e.g., our interview topic guides and text message reminders). Our PPIE-partner (VAF) has contributed to dissemination of study information on social media, through professional networks and charity websites, and worked together with the research team to disseminate project information through national public-facing events such as the “Burning Nights Patient and Carers Conference 2024” (host: VAF; invited speaker: HB; group facilitator: WJC).

Our TAG consists of a group of public contributors who represent organisations with a vested interest in research, policy and/or practice in work and health, digital innovations, or chronic conditions management. The TAG includes four people with lived experience of chronic pain (plus PPIE-partner VAF) who review and guide us on our trial processes, research materials, dissemination and communication plans. Our TSG includes one PPIE-member with lived experience of chronic pain who contributes to oversight of trial procedures and quality assurance. PPIE-members of TAG and TSG preferred not to be identified in a manuscript as individuals with chronic pain.

Overall, the PPIE input through Phase 4 ensures that our research processes and materials are meaningful, relevant, and low burden, and our intervention remains fit-for purpose. All processes in Phase 4 have involved collaboration and shared decision-making with our PPIE-partner (VAF). This feasibility trial ends in November 2025 and will provide insights into the acceptability of the toolkit, people’s views towards it, and how people used it in the context of work. Our learning from Phase 4 PPIE activity will subsequently inform how we engage and work with PPIE-partners, PPIE-members, and PPIE-contributors in the design and delivery of a future definitive trial, and the future scale up and sustainability of our intervention.

### Ethical considerations

As UK-based PPIE and intervention development activity, ethical approval was not required and since PPIE contributors are not research participants they are not required to give formal written consent for their involvement. The PPIE activities in the creation and evaluation of the PAW Toolkit (Phases 1–3) did not require research ethics approval as they were classified as public consultation and educational development by the University of Nottingham Faculty of Medicine and Health Sciences Research Ethics Committee (Ref: FMHS 358–0921; 08 Jan 2020 and 27 September 2021). The same committee subsequently granted research ethics approval for the PAW Toolkit feasibility trial (Ref: FMHS 237–0323; 31 March 2023) – although ethical approval is not required for PPIE activity, this trial approval does include our Phase 4 PPIE plans.

As good practice, across Phases 1–4, our PPIE-partner and public contributors were made aware of the nature of their involvement, their right to withdraw when they wish, any implications and risks of being involved. For synchronous PPIE activity, including the Phase 1 stakeholder consultation event and the Phase 3 concept mapping exercise, two researchers (HB, SS) documented ‘informed verbal consent’ for synchronous activity in the presence of the group. For asynchronous PPIE activity ‘informed assumed consent’ was taken, including Phase 1 anonymous online PPIE surveys with public contributors (employees/employers) and expert peer review (public contributors/stakeholders), Phase 2 expert peer review, and anonymous PPIE evaluation feedback surveys with PAW Toolkit users, and Phase 3 expert peer review. For Phase 2, our PPIE-partner (SG) gathered ‘informed written consent’ from public contributors sharing their views in interviews. In Phase 4, while our PPIE-partner provided verbal consent, written consent is not required as she is considered an equal member of the research team.

## Results of the PPIE

Here, we outline PPIE input at each stage within the research cycle (Phase 1–4) and who was involved in each activity. We describe the impact of PPIE on PAW Toolkit development (Phase 1–3) and the impact of PPIE on the research process (Phase 1–4).

### PPIE input within the research cycle

Here, we describe the ways in which our stakeholders and PPIE-partners, PPIE-members and PPIE-contributors have contributed at every stage of the research cycle (Table 1), at all stages overseen by the lead author (HB) and supported by the broader research team. There were no unsuccessful *routes* to PPIE engagement in this programme of work, although *challenges* in PPIE engagement are reflected upon in the discussion.

**Table 1:** PPIE contributions through the research cycle

Research stage	Route to public involvement and engagement	Who was involved?
**Phase 1: Co-creation**		
Conception: Establishing the support need	Individual consultation Online employer survey Online employee survey	Partners^a, b^/Contributors Partners^a, b^/Contributors Partners^a, b^/Contributors
Conception: establishing the medium as stand-alone / digital	Individual consultation Stakeholder consultation workshop	Partner^a^/Contributors Partner^b^/Contributors
Establishing the theory of change	Individual consultation	Partner^a^/Contributors
Co-creation of content and technical presentation	Individual consultation Stakeholder consultation workshop Expert peer review	Partners^a, b^/Contributors Partner^b^/Contributors Partners^a, b^/Contributors
Role in stakeholder workshop delivery	Stakeholder consultation workshop	Partner^b^/Contributors
Role in sourcing other PPIE reviewers	Expert peer review	Partners^a, b^/Contributors
**Phase 2: Prototype evaluation**		
Exploring views of people with chronic pain towards the PAW Toolkit	Employee survey with toolkit users	Partner^b^/Contributors
Exploring views of professionals with an interest in pain at work, towards the PAW Toolkit	Expert peer review survey with toolkit users	Partner^b^/Contributors
Gathering in-depth insights from people with chronic pain towards their needs at work, and the potential supportive role of the toolkit	Interviews with employees who have chronic pain	Partner^b^/Contributors
Update and refinement of content and technical presentation	Group concept mapping exercise Expert peer review	Partners^a, b^/Contributors Partners^a, b^/Contributors
Ensuring adherence to equality, diversity and inclusion principles	Employee survey with toolkit users Expert peer review survey with toolkit users Toolkit evaluation interviews with employees who have chronic pain	Partners^a, b^/Contributors Partners^a, b^/Contributors Partners^a, b^/Contributors
**Phase 3: Review and update**		
Review and confirmation of the theory of change	Group concept mapping exercise	Partners^a, b^/Contributors
Confirmation of the priorities of people with chronic pain at work and appropriateness of toolkit subsections.	Group concept mapping exercise	Partners^a, b^/Contributors
Refinement of content and technical presentation	Group concept mapping exercise Expert peer review	Partners^a, b^/Contributors Partners^a, b^/Contributors
Ensuring adherence to equality, diversity and inclusion principles	Expert peer review	Partners^a, b^/Contributors
**Phase 4: Feasibility testing**		
Finalising the study design	Individual consultation	Partner^a^/Contributors
Review of protocol and study materials: information sheets, posters, promotional messaging, questionnaires, interview topic guides.	Individual consultation, review and revision	Partner^a^/Contributors
Input into public-facing web materials	Study promotion adverts Project website	Partner^a^/Contributors
Study promotion (web / social media)	Social media posts (e.g., X (formerly Twitter), LinkedIn)	Partner^a^/Members^b^
Advice on routes for recruitment of organisations	Individual consultation (email/meetings) Trial Management Group Trial Advisory Group	Partner^a^/Members^b^ Partner^a^ Partner^a^/Members^b^/Contributors
Advice on plans for maximising employee uptake	Individual consultation Trial Management Group Trial Advisory Group	Partner^a^/Contributors^b^ Partner^a^ Partner^a^/Members^b^/Contributors
Development and review of a series of text message reminders for trial participants	Individual consultation (email/meetings)	Partner^a^/Contributors
Review of written description of opt-in OT support	Individual consultation (email/meetings)	Partner^a^
Membership of Trial Management Group	PPIE-partner attendance at monthly meetings	Partner^a^
Membership of Trial Steering Group	PPIE-member attendance at 6-monthly meetings	Members^a^
Membership of Trial Advisory Group	PPIE-partner and PPIE contributors’ attendance at 3-monthly meetings	Partners^a, b^/Members^b^/Contributors
Project reporting (development work, and feasibility trial)	Individual consultation	Partners^a, b^/Members^b^
Research dissemination	Co-authorship of conference outputs Co-authorship of research articles Joint dissemination at national PPIE events (e.g., Patients and Carers Conference)	Partners^a, b^ Partners^a, b^ Partner^a^
PPIE involvement dissemination	PPIE-partner’s presentation at a National Pain Centre PPIE event at the host university Co-authorship of PPIE case study submitted to a national pain charity	Partner^a^ Partner^a^

From the authorship team: ^a^PPIE-Partner VAF; ^b^PPIE-partner / PPIE-member / PPIE-contributor SG. Note changes to roles in different study phases and over time. PPIE-members and PPIE-contributors are a broader group of members of the public (including stakeholders and people with lived experience of chronic pain) and are not identified individually except for co-author SG

### Impact of PPIE on PAW toolkit development

The final version of the toolkit was co-created in Phases 1–3 (Fig. 2), more details can be found elsewhere [15, 16]. Phase 2 evaluation feedback from the public contributors is provided below, for the expert reviewers (Table 2) and working people living with chronic pain (Table 3).

**Table 2:** PAW toolkit phase 2 expert reviewer evaluation (*n* = 15)

Review Question	Yes / *n*(%)
Is the focus of the resource clear and consistent?	13 (87)
To your knowledge is the information factually correct?	14 (93)
Is the text well written and in short, clear sentences?	13 (87)
Do the suggested links provide the information needed?	14 (93)
Are the broad sections appropriate?	15 (100)
Is the overall presentation appropriate? (e.g. layout, images, links?)	15 (100)
How easy is this resource to access via the link?	15 (100)
Could this be accessed in different settings (e.g. workplace / home)?	15 (100)
Is this package relevant to any employee who has chronic pain?	13 (87)

**Table 3:** PAW toolkit phase 2 public contributor evaluation (*n* = 104)

Question (yes/no)	Yes / *n*(%)
Were you able to access a fully functioning toolkit?^a^	100 (96)
Did you understand the information provided in this toolkit?	101 (97)
Have you gained sufficient knowledge from this resource?	84 (81)
Have you practically used any of the information?^b^	52 (50)
If you have not yet practically used the information, could it have future value for you?	99 (95)
Could this toolkit resource be used by any employee who has chronic pain?	100 (96)
Was engaging with this toolkit time well spent?	96 (92)
Was using this toolkit challenging with regards to your own digital skills?	3 (2)
Did you experience any technical difficulties in using this toolkit?^c^	8 (7)
Was this package appropriate for your needs?	93 (89)
Did this resource contain meaningful information?	103 (99)
Would you recommend this package to a colleague?	100 (96)
**Question (score 1–10)**	**Range**,** Mean**
Was this resource easy to navigate and use? (1 = not at all easy, 10 = extremely easy)	4–10, 8.8
How do you feel about this resource being available for people who have chronic pain? (1 = very negative, 10 = highly positive)	4–10, 9.0
How relevant is this toolkit to people who have chronic pain?^d^ (1 = not at all relevant, 10 = extremely relevant)	1–10, 8.4

Notes: ^a^Those responding ‘no’ reported an employer firewall which blocked access, or mobile phone signal issues. ^b^PAW Toolkit prototype and the evaluation survey were distributed at the same time. ^c^Reported technical difficulties included lack of sound on their own computer/device, an employer firewall which blocked access, or malfunctioning links. ^d^Reasons for lack of relevance primarily related to (1) existing knowledge and good workplace support, (2) not being employed

**Fig. 4:**
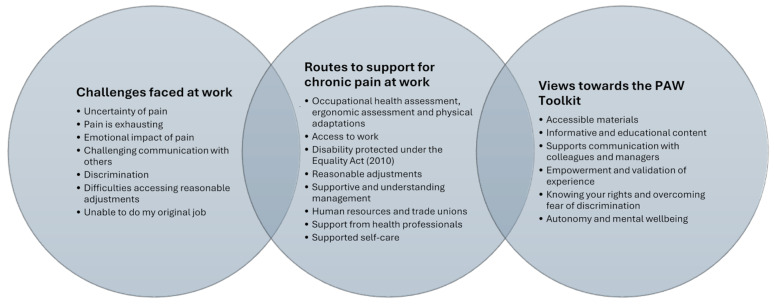
Public contributors’ key points drawn from toolkit evaluation interviews

Our PPIE-partner (SG) categorised key points from the interview discussions with public contributors into three broad areas (Fig. 4): (1) The challenges faced at work, (2) Support accessed in the workplace to manage work alongside pain, (3) Views towards the toolkit.

Further detail about public contributors’ challenges experienced at work, their description of current routes to accessing support in the workplace and illustrative quotes can be found in Additional file 4; these provide the broader contexts of lived experiences in this group that help to inform what types of support the PAW Toolkit needs to provide. Directly aligned with the aim of our PPIE, our PPIE-partner (SG) collated the views of public contributors towards the content, presentation and usability of the PAW Toolkit (as summarised in Fig. 4) which are expanded upon in Additional file 5.

**Fig. 5:**
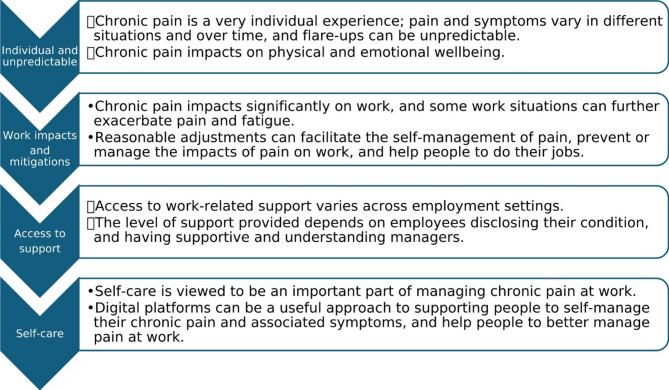
PPIE contributors’ summary of key points within the interview data

Based on these interviews, through discussion, the lead author (HB) and PPIE-partner (SG) created an over-arching summary which highlighted the perceived value, to people with lived experience of chronic pain, of digital interventions to facilitate the self-management of chronic pain at work (Fig. 5). This important view of end-users supports the rationale for the PAW Toolkit and our decision to continue to with updates following its inception and co-creation in 2020.

**Fig. 6:**
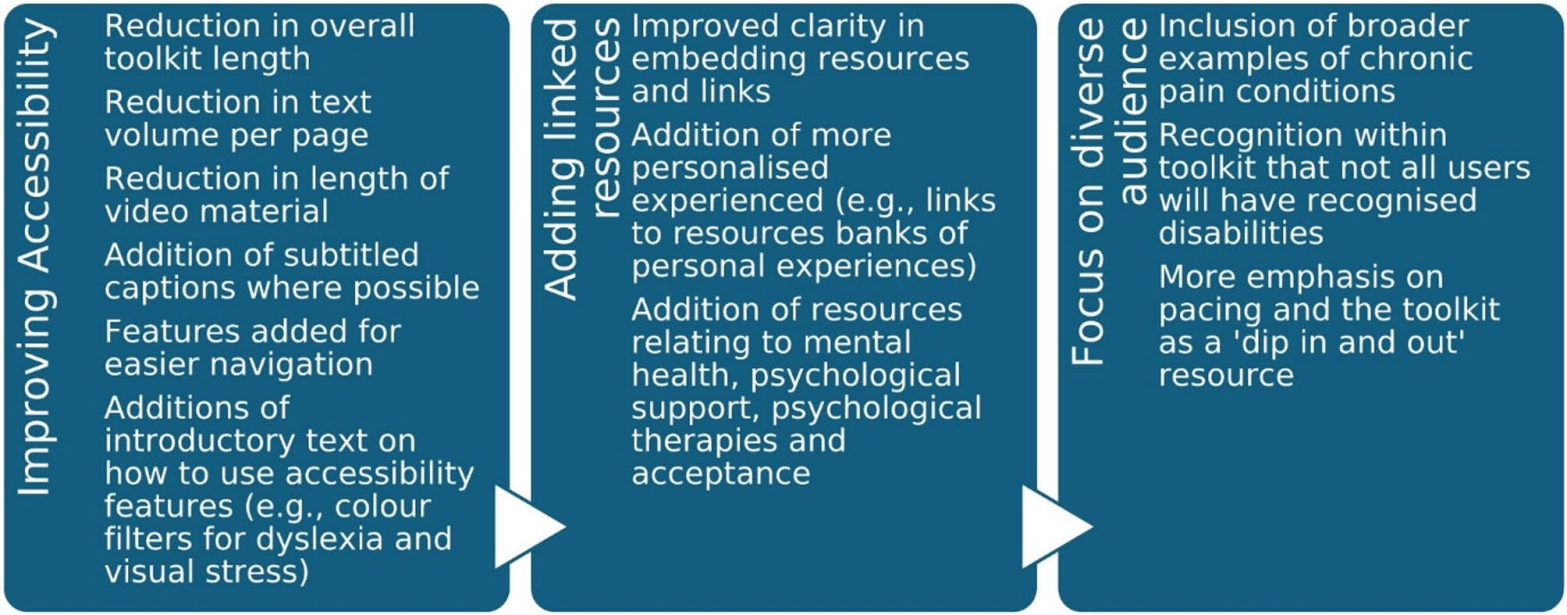
PPIE changes to the toolkit following Phase 2 prototype evaluation

The specific changes made to the toolkit following survey and interview evaluation with public contributors in Phase 2 are shown in Fig. 6. The toolkit revisions were made collaboratively by our PPIE-partner (SG) and the lead author (HB), and the final version was reviewed by a second PPIE-partner (VAF). This process demonstrates not only how PPIE shaped the continued development and update of the intervention (e.g., providing views and guidance), but also how PPIE was embedded within the toolkit production processes (e.g., co-creation).

### Impact of PPIE on the research process

Phase 1 PPIE contributions to PAW Toolkit development co-created the theory of change, content and presentation of the toolkit. This generated a research ‘intervention’ and allowed us to move towards prototype review and evaluation.

Phase 2 PPIE contributions to prototype evaluation established the currency of the toolkit, and its ongoing value. PPIE contributions demonstrated the breadth of individual circumstances, highlighting physical, psychological and organisational challenges faced and a range of support accessed at work (or lack thereof). The toolkit was perceived to be an empowering resource to support employees’ wellbeing, validate their experiences, provide employees with autonomy at work and provide opportunity for advocacy for a collective understanding of chronic pain in the context of work. The toolkit and linked resources were perceived to be accessible, informative and necessary. Toolkit users felt that the toolkit helped to raise awareness of the rights and entitlements of people with chronic pain in the workplace and found that the guidance and resources supported them in communications with others in the workplace about their condition and support needs. They valued the authenticity of resources which shared peoples lived experiences of chronic pain at work, and the breadth of strategies, resources and ideas to support their self-management. This evaluation, with minor recommendations for improvements, allowed us to move forwards to review and update of the toolkit following the COVID-19 pandemic, ready for testing in a feasibility trial.

Phase 3 PPIE contributions to review and update of the PAW Toolkit confirmed the validity of the original theory of change, confirmed that the sections within the toolkit still aligned with the priorities of people with chronic pain over time, and led to a minor update of content and technical presentation which ensured that the toolkit offered advice that was current, and adhered to accessibility guidelines. This allowed us to move forwards to testing the feasibility and acceptability of the toolkit in a feasibility workplace trial.

Phase 4 PPIE partnerships and contributions guided our approaches at every stage of the feasibility trial. We talked to members of the public about our study plans and what would work well, or less well, in their view. We received suggestions about how to recruit organisations to the trial, and how to motivate employees to take part in the research and engage with the toolkit. Our trial PPIE-partner (VAF) has been particularly helpful in supporting us to reach members of the public with our study information. All our project materials were reviewed by our PPIE-partner (VAF), PPIE-members (SG + others) and PPIE-contributors (*n* = 10) to ensure that the language was accessible, and that the information was relevant. This contribution improved the clarity of all our participant-facing materials, including participant and employer information sheets, posters, emails, written and audio-visual website information. PPIE contributions informed the design of our surveys and interview topic guides and informed best practice in approaches to delivery of our intervention and data collection approaches. Embedding a PPIE-partner within our research team (and having PPIE representation all advisory and steering boards) ensures that our regular project meetings are firmly grounded in issues that are important and relevant to people with chronic pain.

Our PPIE-partner co-authors our academic outputs and facilitates lay dissemination which is done in partnership with all members of the research team. This allows us to have confidence that our trial is high quality and is relevant to our target audience.

## Discussion and conclusions

In this section, we discuss components for successful PPIE, and map our Pain-at-Work PPIE conducted over five years to recommended components. We describe specific PPIE challenges arising within our work and management strategies we adopted.

### Components for successful PPIE

In response to concerns about the limitations of checklists and the need for mindful reporting [25], we have gone beyond meeting the minimum requirements of the GRIPP2-SF (simply reporting on checklist items). Rather, we have described in detail, and critically reflected upon, each PPIE stage in separate publications. This allowed for greater depth and mindful reflexive reporting in research outputs relating to our patient and public involvement processes. It has been recommended that future iterations of the GRIPP consider (a) incorporating criteria about whether the checklist is completed by or with service user researchers or not, (b) addressing criteria that position service user research as needing to be justified, and (c) expanding the “critical perspective” element of the checklist to explicitly consider power differentials [25]. We have considered and reported on all three factors here. Regarding (a), we completed the checklist in collaboration with our PPIE-partners from various stages of this work (Phase 1–3: SG, Phase 1–4: VAF) who are both co-authors on this paper. Regarding (b), our reporting relates to extensive public involvement work involving several hundred individuals as public contributors (e.g., employees with chronic pain, employers, healthcare professionals and other stakeholders) undertaken in direct collaboration with our PPIE-partners - therefore we believe this is not confined to justifying the engagement of a PPIE-partner, but rather ensuring that we have appropriately reported on *how* and *when* we have engaged with members of the public and other stakeholders, and the *impacts* of that, in order to inform future research.

Our key reflection is that the single most important factor of the PPIE process is that researchers should deeply and fundamentally value PPIE input. Members of the public should be involved in all aspects of the research from inception to dissemination and this was achieved in Pain-at-Work. We found that building relationships with PPIE-partners and PPIE-members and establishing networks of PPIE-contributors over time helped to ensure that members of the public felt confident to share their views in workshops and meetings. In our view, PPIE contributions to Pain-at-Work led to better decision-making, better-quality research, and ensured that the PAW Toolkit is appropriate and relevant to the audience it is designed for. We actively encourage our PPIE-partner to share their experiences of contributing to this research programme with others (e.g., by contributing to PPIE papers, and giving presentations at PPIE events).

We ensured that PPIE input was appropriately costed in our research. This was essential to ensure that we could work with PPIE contributors in a meaningful way, and value their time as we would any team member. It is challenging to calculate a total ‘cost’ for the PPIE input in this complex programme of work. This is largely due to the scale and duration of the development processes which took place over many years, with differing levels of funding availability. At all times, large numbers of public contributors have volunteered their time at various points due to a vested interest in the subject area with no expectation of financial reimbursement (stakeholders, such as employer representatives, trade union and human resource specialists, healthcare professionals etc.). However, throughout the process, PPIE-partners and contributors with lived experience of chronic pain have been reimbursed for their time and input using ‘payment guidance for researchers and professionals involving people in research’ provided by the UK National Institute for Health and Care Excellence [26].

**Fig. 7:**
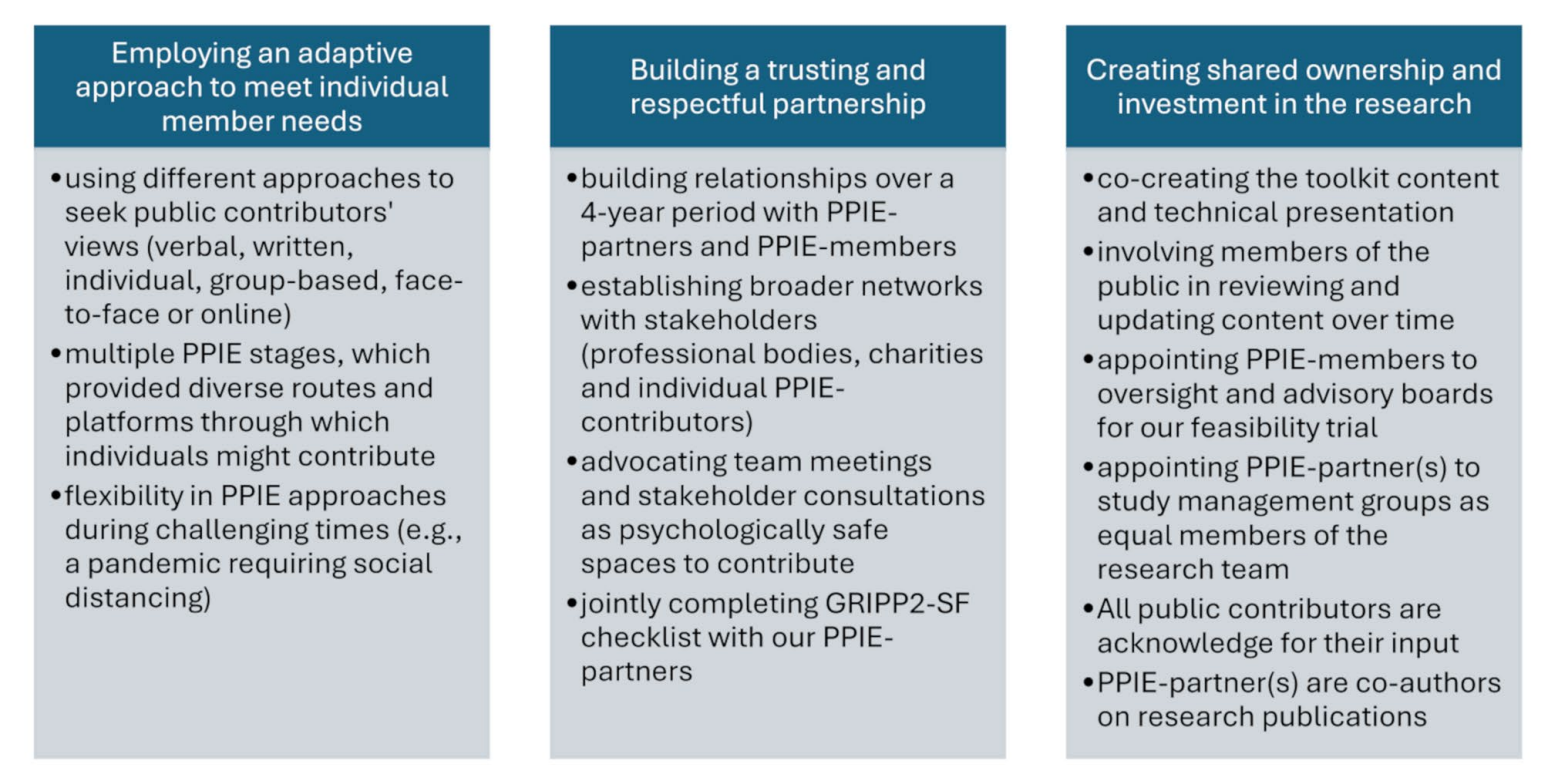
Mapping of Pain-at-Work PPIE to recommended components

Our approach to PPIE is evidence-based, aligning with Pearson and colleagues’ [27] proposed ‘essential components of successful PPIE’ as shown in Fig. 7.

Ensuring there was adequate support for PPIE-partners, PPIE-members and PPIE-contributors was an important component for success. For example, we included a budget for PPIE within our project grants based on INVOLVE guidance, which supported the time of PPIE-partners and PPIE-members for their input (such as meeting or training attendance, review of materials, support with events, collating and summarising views of broader public contributor groups). We had funds to offer vouchers by way of thanks for public contributors involved in various PPIE phases (such as stakeholder consultation, concept mapping exercise, toolkit reviewing). In terms of mentorship, our PPIE-partners and PPIE-members have been supported directly by the academic project lead (HB), and two project researchers (EW, WJC). These team members facilitated the involvement of people with experience of chronic pain in our trial and were the first point of contact for PPIE contributors for clarification of processes or to discuss any project-related concerns. We have worked directly with two national Versus Arthritis funded pain centres, that have PPIE as a core component of their infrastructure. Our PPIE-partner in the feasibility trial is Founder and Chair of Trustees for Burning Nights CRPS Support (a UK-based pain charity), and Chair of the Expert Patient and Carer Committee at the British Pain Society – she has significant experience of PPIE in research. Public contributors identified through this charity were able to access support from our PPIE-partner through peer-to-peer mentoring.

### Challenges and mitigations

A key strength of our study was to consider not only the benefits of PPIE but also the challenges experienced. This is important since few studies report on the more challenging impacts of PPIE, which could represent a publication bias [2]. Challenges were predominantly experienced during group activities occurring at the toolkit development stage. We mapped our PPIE challenges to themes identified in a published systematic review of PPIE activity in health and social care research [2] (Table 4).

**Table 4:** Summary of PPIE challenges and management strategies

Stage of the research cycle	Challenge	Management strategy
**All stages**		
PPIE-partner, PPIE-member and PPIE-contributor recruitment	Difficulty in recruiting a diverse PPIE group. Initial over-representation of individuals with certain characteristics and difficulty reaching seldom heard groups (e.g., relating to gender, job role, organisation type and size, sector). Challenges related to accessibility for PPIE-contributors who had disabilities, or where English was not their first language.	Reached out to potential members via multiple routes including PPIE groups associated with the Versus Arthritis centres, and existing PPIE networks engaged with similar research. Had a high level of support from PPIE-partners for reaching other sectors. Offered recognition and reward (e.g., learning opportunities, formal acknowledgements, financial reimbursement). Budgeted appropriately for PPIE contribution. Ensured accessibility was considered in buildings where face-to-face events took place. Included remote approaches to PPIE contribution (i.e., online meetings, telephone, text) to include those with physical barriers to travel or keyboard use. Translation available where required.
Obtaining PPIE feedback	Some PPIE-members and PPIE-contributors did not contribute any suggestions or changes. Some PPIE-members and PPIE-contributors found it difficult to share their views in online group meetings. Some PPIE-contributors had accessibility issues for face-to-face or online meetings.	Ensured anyone involved in PPIE (partners, members, contributors) know their involvement is valued so they feel confident to contribute. Included PPIE as a standard agenda item in all meetings which was reserved ‘airtime’ for PPIE contributors. Provided multiple routes for individual feedback (such as online meetings, texts, emails and one-to-one in-person or telephone conversations) to engage those who may be less confident in group situations or have accessibility issues. Provided feedback on how PPIE contributions were used.
Relationship with PPIE contributors	Potential for power differentials to affect the communication of genuine views.	Encouraged individuals (PPIE partners, members, contributors) to challenge the status quo. Maximised long-standing relationship with our PPIE-partners who acted as a conduit to reach people with chronic pain and other stakeholders. Multiple routes for feedback which allow avoidance of communicating contrasting views publicly. Contrasting views presented back to the PPIE contributors for consensus building. Provided details of future PPIE opportunities.
Resources: Time	Time burden for the research team associated with engaging PPIE (partners, members, contributors) in planning and conducting research.	Additional time built into the study schedule, and all team members briefed on the importance of PPIE to be able to move to the next research stage.
Resources: Cost	Financial implications of PPIE at every stage of the research cycle.	Appropriately costed for PPIE input using INVOLVE guidance. Targeted research funders that actively encouraged PPIE engagement and supported PPIE budget.
**Intervention design and development**		
Design of the PAW Toolkit – stakeholder consultation event group activities	Difficulty in maintaining PPIE-contributor confidentiality within meetings, where attendees discussed personal experiences.	Maintained a dialogue about confidentiality and professionalism.
Design of the PAW Toolkit – stakeholder consultation event group activities	Groups being dominated by strong characters and their perspectives, leading to over-emphasis on particular issues.	Engaged in careful and inclusive facilitation practices to allow a fair representation of voices and allow conversations to cover diverse issues
Design of the PAW Toolkit – stakeholder consultation event group activities	Groups being overshadowed by personal experience stories, when the aim was to identify and agree on PAW Toolkit content and presentation.	Provided clear instructions for the meeting. Engaged in careful facilitation practices, bringing conversations back to topic.
Design of the PAW Toolkit – stakeholder consultation event group activities	Groups being seen as a forum for those with lived experience of pain to access personal health advice from healthcare professionals in attendance (groups included researchers, clinicians, employment specialists, policy makers).	Communicated clear expectations for the session outlined and careful facilitation practices, bringing conversations back to topic.
Design of the PAW Toolkit – stakeholder consultation event group activities and expert peer review of content and presentation.	PPIE-contributors shared information about the PAW Toolkit content externally prior to completion of the co-creation activity and end of the development phase.	Communicated clear expectations for PPIE involvement and open dialogue about research integrity. Training offered for PPIE partners, members and contributors.
Time-lag between PAW Toolkit design and feasibility testing	A long-lasting global pandemic led to a time-lag between development and feasibility testing which then required additional PPIE work to update and refine the toolkit content. Most original PPIE-contributors were not available.	Conducted a concept mapping study with a new PPIE-contributor group. Long-term relationship with PPIE-partners helped to sustain the connection between the original co-creation activities, PPIE required for the toolkit update and PPIE in the feasibility trial.
**Feasibility cluster randomised controlled trial**		
Design of feasibility trial baseline and follow-up surveys	Difficulty in balancing traditional academic criteria for high-quality research, such as the use of standardised research measures, and PPIE (partner, member, contributor) perspectives.	Reduced unnecessary jargon and text in data collection tools. Reached out to a broader PPIE-contributor group and took the majority view. Compromises were reached to ensure PPIE member views were incorporated in alignment with the research aims.
Membership of Trial Management Group	Potential for lack of clarity regarding whether PPIE-partners are ‘part of’ the research team, or ‘supporting’ the research team.	Assigned a named PPIE-partner to the Trial Management Group, who is considered an equal contributor to the research team and acts as co-author on publications. PPIE-members are external to the trial management – they hold advisory positions through structured groups (Trial Advisory Group, Trial Steering Group) or direct approach as needed (PPIE-contributor groups or individuals)
Dissemination and communication of trial findings	Concerns that PPIE-partners, PPIE-members or PPIE-contributors may disseminate the study results before they have been written up and published in academic journals, thus jeopardising scientific publication.	Maintain open dialogue and communication relating to confidentiality of study findings prior to publication.

## Reflections and critical perspective

In this section, we reflect on the evolving guidance, policies and resources available to researchers around PPIE. We consider some of the key challenges to PPIE (outlined in Table 4) in more depth, and the value of our PPIE beyond the current research programme. We consider the limitations of our PPIE approach. Finally, we provide a summary of a learning with recommendations based on our own experiences that could be considered by other researchers.

### Policies and guidance

In the UK, there are numerous policies, strategies and standards providing guidance on PPIE, often with a focus on collaboration and communication, inclusive opportunities, ethical considerations, governance and impact, safeguarding and confidentiality, and building sustainable relationships. PPIE is, arguably, more established in health and social care compared to other fields. This is largely due to: (a) legal and ethical frameworks (health and social care organisations often have statutory duties to involve the public in decision-making) [28], (b) historical development of PPI through early emphasis on patient safety and service improvement) [29], (c) funding support (many health research funders require PPIE as a condition of funding) [30]. For example, The National Institute for Health and Care Excellence has published a ‘Patient and public involvement policy’ to guide the involvement of lay people, and organisations representing their interests in contributing to developing NICE guidance, advice and quality standards, and supporting their implementation [31]. NHS England has published a ‘Patient and Public Participation Policy’ [32] relevant to the involvement of patients, the public, and NHS staff. The UK Standards for Public Involvement [33] are designed to improve the quality and consistency of public involvement in research. They were produced by the UK Public Involvement Standards Development Partnership which brings together representatives from the Chief Scientist Office (Scotland), Health and Care Research Wales, the Public Health Agency (Northern Ireland) and the National Institute for Health and Care Research (England) working with an independent expert. The partnership showcases experiences from 10 different ‘test bed’ projects which have applied the UK Standards in a range of contexts [33]. Health Data Research UK shares guidance on ‘Involving and engaging patients and the public’, including principles for how to achieve the UK Standards for Public Involvement [33, 34]. The National Institute for Health and Care Research have produced briefing notes for researchers on public involvement in the NHS, health and social care research [35] and The NHS Health Research Authority has published best practice on public involvement in health and care research [36].

PPIE is rapidly involving in other sectors. The UK Research and Innovation (UKRI) is the UK’s national funding agency for research and innovation, supporting research across various disciplines, including science, technology, social sciences, and the arts. It is composed of seven research councils, Innovate UK, and Research England. UKRI has an overarching public engagement strategy [37] which aims to break down barriers between research, innovation and society, through a sense of shared endeavour, supporting collaboration and opportunities and valuing diverse knowledge.

### Reaching under-served groups

Although we had initial challenges in reaching under-served groups, our successful strategies (Table 4) involved working with professional networks and groups, including recognition and reward, appropriate budgeting, addressing accessibility issues and being flexible in our approaches. It is widely accepted that under-served groups are under-represented in research and PPIE [38, 39]. In the UK, considerable efforts have been made to provide detailed guidance on which groups are considered under-served, and how to improve representation of these groups in research [39, 40]. In the NIHR INCLUDE project, Witham and colleagues [40] proposed four key goals to achieving this: building long-term relationships with under-served groups, developing training resources to improve design and delivery of trials for under-served groups, developing infrastructure and systems to support this work and working with funders, regulators and other stakeholders to remove barriers to inclusion. Our development processes reached a diverse group of public contributors overall. We had a diverse group of public contributions in the Phase 1 stakeholder consultation, employer and employee surveys, and expert peer review. In Phase 2 we had a diverse group completing the public and professional evaluation surveys. However, we did not achieve ethnic diversity in our 15 Phase 2 interviews. This may have been impacted by our contributors being sourced by, and interviews being conducted by, a White British PPIE-partner. Future support for PPIE-partners engaging in seeking the view of public contributors in the context of workplace-based research could include (a) more training in ways to actively engage with underrepresented employee groups through targeted outreach and partnerships, (b) having more diverse teams (the researchers and PPIE-partners engaged in seeking public views), and (c) working more closely with employee network groups, such as Black and Minority Ethnic Staff Networks.

A wealth of toolkits is now available to support researchers in increasing the participation of under-served groups in research and PPIE. For example, the NIHR Applied Research Collaboration for the East Midlands provides a toolkit for “Increasing Participation of Black Asian and Minority Ethnic Groups in Health and Social Care Research” [41], the COMET Patient and Public Involvement Toolkit includes strategies for engaging diverse groups in core outcome set studies [42], and the NIHR INCLUDE Project [39] offers a roadmap for improving inclusion of under-served groups in clinical research, with examples of barriers and strategies to overcome them. Our experiences, and these toolkits highlight the importance of building trust through community partnerships (e.g., with local organisations, faith-based groups, and trusted community leaders), having cultural humility and using culturally sensitive approaches, expanding communication channels to widen accessibility, and addressing systematic barriers (e.g., transportation issues, digital access limitations, and consultation fatigue).

### Adapting to unanticipated events

The COVID-19 pandemic occurred in-between developing the toolkit and evaluating it which meant that all our face-to-face PPIE activity moved online due to pandemic-related social restrictions. This was followed by longer-term changes in ways of working for researchers, PPIE-partners, PPIE-members and PPIE-contributors (i.e., hybrid and remote working) and so we retained our online approach. We thought that the lack of face-to-face contact might be challenging, but it worked very well and in fact, made our meetings more accessible to individuals with competing demands on their time, disabilities or barriers to travel. While remote work has challenges, we had management strategies to overcome this (see Table 4). There are key benefits of this approach in terms of lower costs, lower impacts on people’s time, greater flexibility, and less travel for those involved in PPIE which can be important for those living with chronic pain and means that our study has a low environmental impact.

### Maintaining confidentiality

One of our greatest challenges was preventing PPIE contributors from disclosing confidential research plans or findings externally, thus jeopardising scientific publication and our dissemination plans. This required a proactive strategy, involving clear communication at the outset, to set expectations for all involved and explain what information is confidential and why it must be protected. Our activities were GDPR compliant, we shared only information that was relevant and necessary at each phase, and restricted access to sensitive data. PPIE training and support included ethical research practices and the importance of confidentiality. We followed NIHR ethical practice guidelines for public involvement (e.g [43]). Finally, for those members of the public who contributed to more than one phase, we reinforced confidentiality obligations through periodic reminders and discussions.

### Recognising and minimising the impact of power differentials

Power differentials in PPIE refer to the imbalances in influence, authority, and decision-making between researchers, stakeholders (such as healthcare professionals) and members of the public involved in research or policymaking. These disparities can affect the extent to which members of the public feel able to engage and contribute, and ultimately the influence they have in shaping research. Findings ways to address power balances has been identified as an important aspect of making public involvement work [44]. A key aspect of managing power imbalances is to ensure that the perspectives of those with lived experience or formal expertise are valued equally. In our PPIE, this was achieved in several ways. First, through our co-production approach to toolkit development and testing (through inception, content building, technical presentation, and evaluation) and second, by the appointment of PPIE-partners as equal members of the research team. We adopted less hierarchical structures by including members of the public as equal members of advisory and steering boards (PPIE-members) and held events at which diverse stakeholders – such as people with lived experience, employers, and healthcare professionals - engaged in ‘view sharing’ or ‘decision-making’ activities together as equals. While retaining flexibility for individuals to choose to contribute in other ways that avoid communicating in public settings. We sustained relationships with PPIE-partners, built our networks of employer representatives and healthcare professionals over many years, and consistently respected their views. Building equitable partnerships has been identified as an important approach to reducing power imbalances in co-production [45].

### Strengths and limitations of our approach

Our PPIE is perceived to be comprehensive by our PPIE-partners and contributors, and we aimed to be as inclusive as possible in our approaches, genuinely integrating public perspectives and addressing any known barriers to engagement. Co-production and public consultation are the ‘norm’ in our team’s research practices. This is a strength, given concerns about ‘tokenistic’ PPIE practices, whereby PPIE may be conducted superficially as a ‘tick-box’ exercise, or only for reasons of compliance with funder requirements [46]. Despite our efforts to be inclusive, there may be individuals who did not engage in our PPIE for reasons unknown to the research team – this could be a lack of awareness or understanding, study timing, accessibility issues (such as language, culture, disability, or digital exclusion), resource constraints, perceived power imbalances or perceptions of the academic ‘context’ (e.g., as intimidating or complex) [29]. Except for the ‘numbers of contributors’ involved at each stage, we have not presented quantifiable measurements for PPIE such as ‘number of meetings held’. However, this was a conscious decision as we preferred to focus on the breadth and quality of engagement and its influence on research decisions, processes and outcomes.

### Going forwards

Our summary of the learning from our PPIE, and recommendations for other researchers can be found in Table 5. The PPIE input in this programme of research goes beyond the immediate project and helped us to determine the key advantages and challenges of web-based interventions for training and health behaviour change. For example, feedback from PPIE-partners and contributors in our Phase 4 feasibility trial, combined with learnings from other web-based workforce studies, informed the development of the “Web-based Workforce Health Intervention Development and Evaluation Framework” (WWHIDE Framework: [47]) [Additional file 6]. WWHIDE was developed by the lead author and colleagues and is the first framework to present key considerations around the recruitment of employers and employees, intervention design and development, delivery modality, comparison groups for trials, intervention engagement, attrition rates, and user acceptance. Insights from our PPIE-partners and contributors have therefore reached beyond direct input to the trial and have relevance to the design of future health research studies involving web-based interventions for education, training, and behaviour change.

A study exploring the implementation of PPIE across Europe found that PPIE was “not firmly embedded or adequately formalised in European healthcare systems and research” [48].

Given the widely accepted vision that PPIE should be embedded in all health research, we contribute to PPIE practice and the evidence-base in this field. Our worked example of PPIE may serve as a catalyst for other researchers to consider planning, documenting and critically appraising PPIE throughout the research cycle.

While the PAW Toolkit is focused on a health topic (chronic pain), it’s development, evaluating and testing has taken place outside of health and social care (workplaces), albeit involving healthcare professionals as ‘stakeholders’ in expert review and evaluation of content. The application of PPIE in organisational research outside of health and social care is less commonly discussed – and is rarely covered in depth. Our extensive PPIE activity is highly relevant to health and social care researchers and our approaches to PPIE in digital intervention development have broad applicability across health areas. However, the breadth and depth of our work is particularly novel in the context of workplace research, albeit this is an emerging area. Here, we have presented the benefits, challenges and approaches to ‘best practice’ in PPIE in the development and evaluation of an intervention conducted in the workplace settings. Our learning points and recommendations are transferable to other national and international health research contexts and settings.

**Table 5:** Summary of learning points and recommendations

Learning points		
Advantages of PPIE to the Pain-at-Work research	Descriptions	Selected examples
Improved relevance of the toolkit	Our research is better aligned with the needs and priorities of people with chronic pain, making findings more impactful.	Phases 1–3: We co-created the concept, content and technical presentation of the PAW Toolkit and engaged expert peer reviewers throughout the development and update processes (including people with lived experience). Our PPIE processes ensured we were addressing real-world concerns and priorities. Phase 2: Our evaluation confirmed that the toolkit was usable, relevant and appropriate for people living with chronic pain.
Better study design	Public input helped ensure that our studies were designed in a way that is acceptable and understandable to participants	Phase 4: Our PPIE-partners helped to select the outcome measures that were used in our feasibility trial – these reflected the outcomes that were most important to people living with chronic pain.
Enhanced ethical considerations	We were able to identify possible ethical concerns early and come up with solutions.	Phase 4: Our feasibility trial was designed so that individual employees did not have to disclose their health conditions or their participation in the feasibility trial to their employer. No health data was shared with employers by the research team. The digital intervention (PAW Toolkit) and study materials adhered to accessibility guidelines.
Better recruitment and retention	We were able to find the best routes to reaching organisations of different types, sizes and sectors. We identified the most appropriate routes to recruiting and retaining employees in a trial.	Phase 4: PPIE improved our outreach to organisations and employees for recruitment, by suggesting specific professional networks and organisations to approach. We acted on suggestions for reaching a diverse research sample in our feasibility trial, such as working with Trusted community leaders and engaging with staff networks (e.g., Black and Minority Ethnic Staff, Disabled Staff). We enhanced accessibility (of information and the research team) by simplifying information sheets and website materials and providing alternative forms of communication. Text messaging reminders and an opt-in prize draw incentive were proposed to maximise retention. The evidence of success is that we exceeded recruitment targets. The feasibility trial included 18 organisations (50% over target) and 380 employees (217% over-target). Both our recruitment and retention rates demonstrate feasibility of the trial processes.
Clearer communication	PPIE ensured that our participant information for research participants was understandable, improving informed consent and engagement. Findings were more effectively shared with participants and the wider public, improving transparency	Phase 1–4: People with lived experience contributed to our website information, study promotional materials, participation information sheets and consent forms, lay summaries of findings, PPIE ‘case studies’ of best practice, and research outputs. Our PPIE-partners were involved in written and oral dissemination.
Higher-quality research	Involving the public led to a better-designed and conducted study, and more meaningful research.	Post Phases 1–4: The research team and PPIE-partners critically reflected on the process and outcomes using the GRIPP2-SF. The PPIE is reported here with detail and transparency.
**Recommendations for researchers embarking on PPIE**		
Involve the Right People	Engage individuals with relevant lived experience to ensure the research is meaningful and impactful. Involve other key stakeholders with a vested interest in the subject area, service delivery, commissioning or supporting those with lived experience.	
Early and Continuous Engagement	Involve the public from conception and design stage through to dissemination to improve study relevance and explore ethical considerations. Build relationships and networks with the public over time.	
Clear Communication	Clearly outline expectations, including time commitment, project duration, and the value of PPIE input. Make research findings accessible and understandable to participants and the wider public.	
Two-Way Interaction	Public involvement should be more than just informing; it should allow for feedback and collaboration.	
Flexible approaches	Use a range of methods such as individual or group discussion, peer review panels, interactive workshops, surveys, videocall and email communications.	
Ethical considerations	Ensure ethical approval is obtained when necessary and differentiate between research and PPIE activities.	
Adequate Support and Resources	Provide training and resources for public contributors to ensure their involvement is effective and valued.	
Reflection and transparency	Use a framework to document and evaluate PPIE activity. Be transparent about the processes and decision-making. Evaluate how PPIE has influenced research design, methodology, data analysis, and dissemination. Assess whether PPIE has led to more person-centred research, improved accessibility, and greater public engagement over time. Reflect on the benefits and challenges together with PPIE contributors. Share learning with other researchers and the public.	

## Electronic supplementary material

Below is the link to the electronic supplementary material.

**Supplementary Material 1**: **Additional file 1**: GRIPP2 short form.

**Supplementary Material 2**: **Additional file 2**: PAW Toolkit PPIE Evaluation Survey.

**Supplementary Material 3**: **Additional file 3**: Characteristics of Phase 2 public contributors (*n* = 104).

**Supplementary Material 4**: **Additional file 4**: Challenges and routes to support.

**Supplementary Material 5**: **Additional file 5**: Public contributors’ views towards the PAW Toolkit.

**Supplementary Material 6**: **Additional file 6**: WWHIDE Framework.

## Data Availability

Data is provided within the manuscript or supplementary information files.

## Abbreviations

GRIPP: Guidance for reporting involvement of patients and the public
GRIPP2-SF: Guidance for reporting involvement of patients and the public 2 short form
PAW: Pain-at-work
PPIE: Patient and public involvement and engagement
SME: Small-to-medium sized enterprise
WWHIDE: Web-based workforce health intervention development and evaluation
