# Supplementary material for: Five years of patient and public involvement and engagement (PPIE) in the development and evaluation of the Pain-at-Work toolkit to support employees’ self-management of chronic pain at work
Source: Res Involv Engagem. 2025 Jul 15;11:81. doi: 10.1186/s40900-025-00757-5 (PMC12261548; doi:10.1186/s40900-025-00757-5)
Supplement: Supplementary file 1 — Supplementary Material 1: Additional file 1: GRIPP2 short form. [file 40900_2025_757_MOESM1_ESM.docx]

**Additional file 1:** GRIPP2 short form

| **Section and topic** | **Item** | **Reported on page** |
| --- | --- | --- |
| 1: Aim | Report the aim of PPI in the study | 5-6 |
| 2: Methods | Provide a clear description of the methods used for PPI in the study | 6-24 |
| 3: Study results | Outcomes—Report the results of PPI in the study, including both positive and negative outcomes | 25-26 |
| 4: Discussion and conclusions | Outcomes—Comment on the extent to which PPI influenced the study overall. Describe positive and negative effects | 26-28 |
| 5: Reflections/critical perspective | Comment critically on the study, reflecting on the things that went well and those that did not, so others can learn from this experience | 28-34 |

*PPI* patient and public involvement
