# Supplementary material for: Five years of patient and public involvement and engagement (PPIE) in the development and evaluation of the Pain-at-Work toolkit to support employees’ self-management of chronic pain at work
Source: Res Involv Engagem. 2025 Jul 15;11:81. doi: 10.1186/s40900-025-00757-5 (PMC12261548; doi:10.1186/s40900-025-00757-5)
Supplement: Supplementary file 2 — Supplementary Material 2: Additional file 2: PAW Toolkit PPIE Evaluation Survey. [file 40900_2025_757_MOESM2_ESM.docx]

**Additional file 2.** PAW Toolkit PPIE Evaluation Survey

Please tick a response for each item, then ideally expand on your answer with a comment.

Question 1: Is revision to Version 1.0 of this toolkit (package) required?

Yes____No ____

Overall Comment:

Question 2: Is the focus of the resource clear and consistent?

Yes____No ____

Comment:

Question 3: To your knowledge is the information factually correct?

Yes____No ____

Comment:

Question 4: Is the text well written and in short, clear sentences?

Yes____No ____

Comment:

Question 5: Do the suggested links provide the information needed?

Yes____No ____

Comment: Are there any other links to additional resources that you could suggest?

Question 6: Are the broad sections appropriate?

Yes____No ____

Comment: Is there anything else that should be included?

Question 7: Is the overall presentation appropriate? (e.g. layout, images, links?)

Yes____No ____

Comment:

Question 8: How easy is this resource to access via the link? (please tick)

Easy to access_______ Not easy to access__________

Question 9: Could this be accessed in different settings (e.g. workplace / home)

Yes____No ____

Comment:

Question 10: Is this package relevant to any employee who has chronic or persistent pain?

Yes____No ____

Comment:

Thank you for completing this form.
