# Supplementary material for: Five years of patient and public involvement and engagement (PPIE) in the development and evaluation of the Pain-at-Work toolkit to support employees’ self-management of chronic pain at work
Source: Res Involv Engagem. 2025 Jul 15;11:81. doi: 10.1186/s40900-025-00757-5 (PMC12261548; doi:10.1186/s40900-025-00757-5)
Supplement: Supplementary file 3 — Supplementary Material 3: Additional file 3: Characteristics of Phase 2 public contributors (n = 104). [file 40900_2025_757_MOESM3_ESM.docx]

**Additional file 3.** Characteristics of Phase 2 public contributors (n=104)

| **Characteristic** | **n(%)** |
| --- | --- |
| Age group  18-24  25-34  35-44  45-54  55-64  65+ | 6 (6)  10 (9)  28 (27)  35 (34)  23 (22)  2 (2) |
| Gender  Female  Male  Non-binary | 87 (84)  12 (11)  5 (5) |
| Ethnicity  Arab  Asian/Pakistani  Asian/Indian  Black African  Black Carribean  Mixed-White/Black Carribean  Mixed-Other  White British  White Irish  White Other  Preferred not to say | 1 (1)  3 (3)  4 (4)  3 (3)  2 (2)  1 (1)  0  77 (74)  3 (3)  9 (8)  1 (1) |
| Employment status  Full-time  Part-time  Long-term absent from work | 54 (52)  47 (45)  3 (3) |
| Organisation type  Large (250+ employees)  Medium (50-250 employees)  Small (10-49 employees)  Micro (1-9 employees)  Self-employed  Preferred not to say | 57 (55)  10 (10)  11 (10)  3 (3)  7 (7)  16 (15) |
| Sector  Public sector  Private sector  Voluntary sector  Preferred not to say | 55 (53)  24 (23)  10 (10)  15 (14) |
