# Supplementary material for: Five years of patient and public involvement and engagement (PPIE) in the development and evaluation of the Pain-at-Work toolkit to support employees’ self-management of chronic pain at work
Source: Res Involv Engagem. 2025 Jul 15;11:81. doi: 10.1186/s40900-025-00757-5 (PMC12261548; doi:10.1186/s40900-025-00757-5)
Supplement: Supplementary file 4 — Supplementary Material 4: Additional file 4: Challenges and routes to support. [file 40900_2025_757_MOESM4_ESM.docx]

**Additional file 4:** Challenges and routes to support

| **“Challenges faced at work”**  Participants’ experiences of work varied according to their health condition and its impacts, the nature of their job role, the workplace environment, and prior experiences of accessing support within their organisation. | |
| --- | --- |
| 1. *Uncertainty of pain*   Participants discussed the unpredictable nature of chronic pain. Learning to understand and then manage fluctuating symptoms and flare-ups presented significant difficulties for individuals. The more recent a participant’s chronic pain condition, the more difficult this was, as they reported having limited understanding of their pain and had less knowledge and experience of using different coping strategies. Participants expressed their physical discomfort at work and described how fluctuating symptoms and intense pain levels affected them. Pain levels varied according to the tasks undertaken during the working day. Periods of standing, for example, or having to lift or move objects, could increase pain levels the subsequent day. | *“…there's no rhyme or reason to it...it can just come on whenever, and I think that's probably been one of the biggest challenges that I’ve faced (ID2:* female, large public sector)  *“…how your pain can be in the morning, it might not be that bad, but 3 hours later… you're in agony (ID7:* female, large public sector)  “*(I may be) fine at the time (but) pay for it the next day (ID12:* female, large public sector). |
| 1. *Pain is exhausting*   Participants spoke about the exhaustion they experienced with chronic pain, and how this affected their work. They experienced challenges in keeping up with workloads, and work-related stress could exacerbate their pain. Participants described feelings of exhaustion from the effort involved in trying to find solutions to their pain and the impact of fatigue and poor concentration on their work and interactions with colleagues. One participant spoke about the interrelated factors of their pain levels, lack of sleep and the impact of medication.  Several participants spoke about exhaustion associated with the extra demands of commuting and travelling to work. For many participants, this challenge had been eased by the transition to remote working due to the COVID-19 pandemic. | *“People think you’re not listening and you’re trying so hard to concentrate, but can’t process anymore”* (ID7: female, large public sector).  *“When you are in constant pain, I think fatigue is a massive issue because when you're working full time, you feel absolutely exhausted* (ID7: female, large public sector).  *“...like walking through treacle” (ID11: female, large public sector)* |
| 1. *Emotional impact of pain*   Many participants spoke about the anxiety they experienced through efforts to manage their pain at work, the practicalities of doing their job with chronic pain, and negative impacts on their mental health.  One participant spoke of how debilitating their pain was and how it negatively affected their confidence and self-esteem. Another participant highlighted the isolation they felt after needing to relocate office away from their team due to their mobility issues and the absence of an office lift to enable them to work upstairs. | *“…an area that people find hard to get, that pain isn’t just physical* (ID9: female, long-term absent).  *“I feel broken by it, I feel a sense of shame and failure and inadequacy* (ID13: female, medium private sector)**.**  *“It left me and a few of my colleagues isolated in what was our old training room, 'cause I was using the wheelchair (ID15:* male, large private sector). |
| 1. *Challenging communication with others*   Many participants described difficulties in communicating with colleagues about their condition, particularly their line managers. A perceived lack of understanding was common; this lack of awareness could lead to situations that exacerbated their pain.  When describing their intermittent pain and fluctuating symptoms, many participants felt judged and disbelieved by others which made conversations about pain and work more difficult. This was a particular challenge for those with less visible disabilities or health conditions. Younger participants felt judged and disbelieved by older colleagues with relation to their ability to undertake certain tasks. | *“Other people booking back-to-back meetings without rests for me, or time to get to the bathroom* (ID15: male, large private sector).  *“...feeling that people don’t believe me, feeling I need to justify myself...people ‘imply’ they don’t believe you - by asking for more evidence (ID2:* female, large public sector).  *“...older colleagues often go..oh (you) should be doing this more physical task* (ID6: female, large private sector). |
| 1. *Discrimination*   Some participants had experienced discrimination towards them in their workplace and had elicited support from Human Resources and/or trade unions. Other participants were apprehensive to disclose their health condition due to the fear of discrimination they may face, including fear of being made redundant or not getting a job if employers knew. | *“People fear being marginalised or targeted”* (ID12: female, large public sector).  *“They tried to get me discharged on medical grounds ‘cause they said I couldn’t do the job, even though I was doing the job. I had a bit of a rough time”.* (ID8: female, large public sector). |
| 1. *Difficulties accessing reasonable adjustments*   Several participants spoke about their difficulties in accessing reasonable adjustments. Experiences were varied but often, individuals were not aware that reasonable adjustments were available at their workplace, whether they were entitled to them or what types of support they could request. Options for support were generally not made available to them by their employer. | *“I didn’t know what I was entitled to or who I could turn to* (ID4: female, large public sector).  *“If I’d had this (toolkit) given to me, maybe I’d have been in a much stronger position to help myself”* (ID9: female, long-term absent). |
| 1. *Unable to do my original job*   Most of the participants were able to continue in their original jobs, with varying experiences of accessing adjustments. However, there were some participants who had been unable to return to their original job and had subsequently experienced redundancy or a change in job role. For example, one participant on their return to work couldn’t do many of the original tasks, was put on a ‘risk register’ and faced redundancy. | *“I managed to secure a more suitable job, but it was right at the eleventh hour, before I was basically sacked”* (ID14: female, large public sector |
| **“Routes to support for chronic pain at work”**  A variety of support had been accessed by participants and was perceived to be beneficial to their ability to manage work alongside their pain. | |
| 1. *Occupational health assessment, ergonomic assessment and physical adaptations.*   More than half of the interviewees, all from larger organisations, had accessed physical adaptations in the form of supportive equipment, including sit/stand desk, exercise balls (to sit on, for alleviation of back pain), split keyboard, adapted mouse and computer screens. Participants had accessed equipment through formal occupational health assessment; some had additional follow-on ergonomic assessments of their working environment. | *“I was sent to occupational health, got some equipment eventually and that was helpful, but I had to wait a long time”* (ID8: female, large public sector). |
| 1. *Access to Work scheme*   Three participants had accessed support through the Access to Work scheme. This is a publicly funded employment support programme in the UK that aims to help disabled people to start or remain in work through the provision of practical or financial support. These participants had received an assessment and recommendations for their employers, and/or funding to support work adjustments, including travel costs. | *“support with taxi costs”; “funded some equipment”* |
| 1. *Disability protected under Equality Act 2010*   Most of the participants had not disclosed a disability or did not refer to themselves as disabled. However, it was recognised that disability disclosure may facilitate access to support. One participant recognised that their disability was protected under the Equality Act 2010 and highlighted how they felt confident, as a result, to access occupational health support when they needed it, and not just if they had been absent from work. Participants acknowledged a wider issue of the challenges faced by many in recognising and/or accepting their health condition in terms of a disability. | *“I think there are challenges out there in terms of accepting the label of a disability” (ID6: female, large private sector).*  *“I must admit I've been struggling to think of it as a disability, and it's taken quite a few people pointing out that yes, it is…and the idea of coming to terms with the fact that this is going to change how I live my life (ID4: female, large public sector).*  *“a lot of people don’t think they’re disabled because they’ve got pain* (ID8: female, large public sector). |
| 1. Reasonable adjustments   Participants described a range of reasonable adjustments they had accessed through their employer; many were recipients of specific equipment or physical adaptations. Some participants were able to work from home which helped them to manage their chronic pain and enhanced productivity. A few participants who had not previously been able to work remotely benefited from the social restrictions during the COVID-19 pandemic which enforced home working during specific periods. All participants who were able to work from home experienced a positive impact on their health and a reduction in pain. This was associated with reduced travel to and from work, being able to manage their own work schedules, and working in a more comfortable environment. Participants spoke positively about other reasonable adjustments to their work, including reduced hours or altered work patterns, risk assessments and flare-up plans (where employees had control over their management), and time off for medical appointments which was not counted as sick leave. Four participants referred to support they received for travel, including special parking arrangements provided by their employer, costs for taxi travel supported by Access to Work, and a ‘motability’ car supported by government funding. The PAW Toolkit had catalysed some participants’ understanding of reasonable adjustments. | “Helps people to understand reasonable adjustments – many people don’t understand what they are”. (ID8: female, large public sector).  “I like the positives about what you should speak to your employer about” (ID3: male, large private sector). |
| 1. *Supportive and understanding managers*   Having a supportive manager was cited as being critical to effectively self-managing chronic pain at work. Some employees had managers who were supportive and understanding, whereas others felt their managers lacked awareness of managing a chronic condition at work and were therefore less likely to offer support. Several participants highlighted the role managers played in facilitating open discussions about health and work challenges and helping them to access support which would enable them to do their job. | “*being able to be open with them…they also have chronic illness and their understanding really helps”* (ID6: female, large private sector).  *“my boss is really understanding, so if I do get pain flares and I can't focus, he's fine with me…resting, getting myself back together again and then rejoining the workforce.* *The company’s been extremely supportive” (ID3: male, large private sector).* |
| 1. *Human resources and trade unions*   Four female participants reported experiencing disability discrimination in the workplace related to their pain condition. In the absence of support from managers and/or colleagues, they reported the support they had received from human resources and trade unions as ‘invaluable’. While there were only three male participants, none reported experiencing discrimination. | *“If I hadn't received the support I did from these people, I think I would have given up a long time ago, 'cause you don't always know what rights you have.* (ID7: female, large public sector). |
| 1. *Support from health professionals*   Access to support from healthcare professionals was through a mixture of in-house services (i.e., via the employer), NHS or private health referrals. Six participants had previously, or were currently, accessing support for pain management through secondary care services, such as NHS pain clinics or structured pain management programmes. Peer support in pain management programmes was perceived to be valuable in helping people to manage their pain. Participants commented on a range of therapies they had accessed from healthcare professionals, including physiotherapists, occupational therapists, and psychologists. Two participants spoke about the benefits of formal psychological therapies, particularly cognitive-behavioural therapies. Some participants had accessed alternative therapies such as chiropractic and osteopathy. Most of the participants spoke about prescribed pain medication, although comments were both positive (i.e., in terms of pain reduction to be able to work) and negative (i.e., in terms of experiencing side effects which would impact further on work). | “*I've had to come off the morphine because I could not function at my job…which is quite high level” (ID11: female, large public sector).*  *“I try not to use meds as I don’t want to be in a drug-induced stupor all the time… but covid has impacted my normal coping strategies...so I’m back on strong meds now” (ID8: female, large public sector).* |
| 1. *Supported self-care*   Several participants described using self-care techniques and tools to support their pain management, such as practising mindfulness, engaging in strategies for sleep management and taking a different approach to managing work tasks. These were perceived to impact on their ability to function at work. Digital resources were commonly used, such as Apps (i.e., *Headspace*, *Curable*) and web-based education or support (i.e., *Breathworks* *Mindfulness for Health*). | *“pacing and breaking things up into smaller chunks is helpful advice I’m trying to do this* (ID4: female, large public sector).  *“[the toolkit] helped* me view goal setting in a different way, more achievable” *(ID15: male, large private sector).*  *“[the toolkit] helped me look into managing fatigue...i didn’t realise there was anything you could do about it before”* (ID7: female, large public sector).  *“The Curable app, for self-management gave me the structure to work with the pain” (ID9: female long-term absent).*  “*It [Breathworks* *Mindfulness for Health] touched on everything that I come up against from a pain point of view, you know it's lack of sleep, it's fatigue, It's all the things that you wouldn't really associate with pain directly, so it's all the other stuff that goes with it, which stresses you out…and mindfulness is helping me deal with that”. (ID15: male, large private sector).* |
