# Supplementary material for: Five years of patient and public involvement and engagement (PPIE) in the development and evaluation of the Pain-at-Work toolkit to support employees’ self-management of chronic pain at work
Source: Res Involv Engagem. 2025 Jul 15;11:81. doi: 10.1186/s40900-025-00757-5 (PMC12261548; doi:10.1186/s40900-025-00757-5)
Supplement: Supplementary file 5 — Supplementary Material 5: Additional file 5: Public contributors’ views towards the PAW Toolkit. [file 40900_2025_757_MOESM5_ESM.docx]

**Additional file 5.** Public contributors’ views towards the PAW Toolkit

| 1. *Accessible materials*   Overall, most of the public contributors considered the PAW Toolkit to be easy to use and to navigate, with clear, simple and useful resources. Participants valued the combination of text, animation and videos. Short, simple text was appreciated by readers with chronic pain, who reported frequent difficulties with concentration and fatigue that could impact on their engagement with reading material. A minority considered the toolkit too long and “too wordy” in places. Suggestions were made for condensing the text, including more personalised examples and improving accessibility features, particularly caption facilities and shorter length videos. |
| --- |
| 1. *Informative and educational content*   All public contributors welcomed the PAW Toolkit and appreciated the underlying need for it among people with chronic pain: *“It’s a much-needed toolkit”* (ID11: female, large private sector); “*It’s an awesome thing”* (ID9: female, long-term absent).  Given the diversity in pain experiences and people’s support needs, it was perceived that the generic content of the resource was useful and preferable to a condition-specific toolkit; the latter was thought to be restrictive and less wide-reaching across those living (and working) with chronic pain. Individuals considered the toolkit to be informative and useful, raising awareness of the support available to people at work.  *“...as somebody who has actively looked for these things before, there's a lot of stuff in there that I hadn't seen before, so that was good (ID6: female, large private sector).*  *“Describes chronic pain well, helps understanding” (ID15: male, large private sector).*  *“really good to have available – you don’t know what you don’t know (ID9: female, long-term absent).*  *“It is a really good resource and I do wish that at the start of my journey, if this kind of thing had been available, I feel like I would have been a lot better informed… there are things in there that I've not heard of before… so, it is still a good resource, even as somebody who's quite far along in my journey (ID6: female, large private sector).*  The high level of input from PPIE contributors in the toolkit development process was valued and seen to support the validity of the resource content.  *“Really important that people with chronic pain have been consulted throughout, design stage to evaluation”* (ID11: female, large private sector). |
| 1. *Supports communication with colleagues and managers*   Most of the public contributors discussed the value of the PAW Toolkit in supporting communication with employers and colleagues about their condition, largely relating to the impacts of pain on work, and approaches to accessing support in the workplace.  “*Really good, useful tool to go through with employer, helps with explaining; this is what my pain is like and (to) realise there are different strategies to help”* (ID12: female, large public sector).  Given the high prevalence of chronic pain in the population, some individuals suggested that the toolkit could be used not only to support individual staff members, but also to raise awareness about chronic pain across the organisation by including it in staff training provisions, or manager inductions. |
| 1. *Empowerment and validation of experience*   The most common response from public contributors was sense of empowerment they felt after accessing the toolkit. Individuals commented on the toolkit’s role in validating their experiences and helping them to recognise they were not alone in their experience. The factor perceived to be of greatest importance was that employees who were ‘not heard’ by managers, felt they would be believed in their communications with employers.  *“It helps to explain my challenges...I’m fed up of explaining things, it can be quite upsetting”* (ID7: female, large public sector).  *“If I’d had something like this on returning to work, I would feel more empowered and in control”* (ID14: female, large public sector).  *“…a toolkit like this validates these experiences, they aren't just personalised for one individual… they are experienced by many people. … makes them feel like they're not alone…and that helps with mental health” (ID7: female, large public sector).* |
| 1. *Knowing your rights and overcoming fear of discrimination*   Most of the public contributors considered the information presented in the toolkit to be significant in helping people to understand their rights as an employee with a disability or chronic health condition, and their entitlements to support options. It was suggested that the toolkit could encourage other people to overcome fear of discrimination and disclose their condition to their employer, and that it might be a catalyst for helping people to access support across the workforce.  *“I didn’t know what my rights were before looking at this...and have used the toolkit for support*” (ID2: female, large public sector).  *“…the legislation around the Equality Act and employees with disabilities is really quite significant in terms of people's rights, that helped me to take action with HR, unions* (ID4: female, large public sector). |
| 1. *Autonomy and mental wellbeing*   Several public contributors emphasised how important it was for people to use the toolkit in their own time, and also over time, when they were ready to engage with it. The toolkit was commended for messaging advocating that users could access and return to the toolkit whenever they are ready to and when different ideas resonate with them. The information was considered by some, as not easy to digest all at once, depending on where people were at with their pain journey.  *“You’ve got to be ready to hear...and you have to keep on saying it, so having something like this available for people to keep dipping into...it’s helpful” (ID9: female, long-term absent).*  The sense of autonomy participants experienced in having control over when and how they accessed the toolkit was perceived to have a positive influence on mental wellbeing.  *“You don't necessarily have to use all of it, but you can use aspects of it to help support your well-being…and I think having a certain level of control will support your mental health” (ID14: female, large public sector).* |
