# Supplementary material for: Five years of patient and public involvement and engagement (PPIE) in the development and evaluation of the Pain-at-Work toolkit to support employees’ self-management of chronic pain at work
Source: Res Involv Engagem. 2025 Jul 15;11:81. doi: 10.1186/s40900-025-00757-5 (PMC12261548; doi:10.1186/s40900-025-00757-5)
Supplement: Supplementary file 6 — Supplementary Material 6: Additional file 6: WWHIDE Framework. [file 40900_2025_757_MOESM6_ESM.docx]

**Additional file 6:** The WWHIDE Framework

**The WWHIDE Framework: A Web-based Workforce Health Intervention Development and Evaluation Framework.**

| Framework components | Items for consideration |
| --- | --- |
| Intervention design and development | - Is this a needs-based intervention? Did the intervention arise from users’ needs? Or address a gap identified by end-users? - Has there been patient and public engagement activity? Was the intervention developed with input from, or co-created with end-users and stakeholders? - Has content been externally peer reviewed? - What are the pre-determined dose parameters (e.g., duration, and frequency) of the intervention? - Is the intervention based on a Theory of Change? - Does the intervention have any theoretical underpinning? Is the intervention informed by theory or theories? (e.g., behaviour change theories, communications theory). - Is the intervention evidence-based? Is the content informed by relevant research? - Does the intervention content align with national and/or international guidelines and policies? - Does the intervention incorporate good pedagogical design practices? - Does the intervention incorporate other principles that inform content design (e.g., persuasive systems design, behaviour change principles)? - Does the intervention adhere to current accessibility guidelines? Is it inclusive for people with disabilities or barriers to learning? - What language is the intervention developed in? Is it available in other languages or formats? - What type of media does the intervention include? (e.g., brief text, images, multimedia, hyperlinks). - Are logos for the institutions that developed the intervention and funded its development visible to end-users to demonstrate credibility and trustworthiness? - Are those individuals and organisations who were involved in the development given appropriate credit (i.e., named within the web-based intervention?) - Are there strategies in place to maximise end-user engagement with the intervention? (e.g., flexibility for access, low technological skill requirement). - What approach is used to communicate information and/or behaviour change principles? (e.g., direct instruction, experiential learning). - Is the content perceived to be relevant by end-users? - Is the content and format acceptable to end-users? |
| Delivery modality | - Is the intervention stand-alone or designed to be used alongside another intervention or with support? - Is the web-based intervention adjunctive to another treatment? (i.e., to supplement face-to-face intervention or print materials). Or is another treatment modality adjunctive to the web-based intervention? (i.e., web-based supported by email, text, or telephone support). - If there is an adjunctive treatment, what is the purpose, duration, and frequency, and how will uptake and engagement be documented? (e.g., this may be to reinforce material, facilitate skill acquisition or initiate behaviour change, provide additional support, enhance motivation and engagement). - Who will deliver the intervention? Is it automated or does it involve support? (individual or group). - Is training required for the intervention delivery (or its use)? - How is the intervention accessed? - Is the web-based intervention opt-in or opt-out? - Is the adjunctive treatment modality (if relevant) opt-in or opt-out? - Is delivery of the web-based intervention synchronous or asynchronous? - Does the intervention cost anything to access? Will participants be paid to access it during the trial? - Can participants access the intervention in their workplace / during their working hours? - Is technical support available throughout the trial to resolve any arising technical issues? - Will participants be sent reminders to access and engage with the intervention? What will be the content, frequency and duration of reminders and how will this be determined? |
| Intervention engagement | - What efforts will be made to maximise the engagement of end-users with the intervention? - How will intervention engagement be measured? - What are the defining active ingredients of the web-based intervention and/or any components of the intervention (to be able to discern intervention effects)? How will this be measured or explored? |
| Research design | - How will the web-based intervention be evaluated? - Will the intervention be tested in a trial (feasibility, acceptability, effectiveness)? - For trials, what will the unit of randomisation be? Organisations or individual participants? Who will conduct randomisation? - Will control or comparison conditions be utilised? - What is the allocation ratio? Is there any blinding to group allocation? - Where relevant, are organisations and participants willing to be randomly allocated to groups? How will this be measured? - Where relevant, has the risk of contamination been minimised? How will this be achieved? - How is assessment of outcome being undertaken? Objective or subjective measures, paper-based or online? - Are outcome measures assessed independently of intervention delivery? - How will delivery of the intervention be implemented? - How will delivery of the intervention be monitored? - What are the inclusion and exclusion criteria? For both organisations and individual participants. - What are the target sectors and organisations? Are organisations small-to-medium sized enterprises, large organisations, or both? - Who are the target participants? What is their work status, occupation, work pattern? What information will be recorded? (e.g., age, gender, ethnicity, job role or job type). - Will settings and participants be heterogenous (through minimising exclusion/inclusion criteria) and if so, what information will be documented? - What data will be collected for participants and organisations? - How will data be collected (e.g., interviews, focus groups, surveys - paper-based or online)? - Is data self-reported and/or from organisational records? - Will there be any data captured on the cost-effectiveness of the intervention? |
| Comparison group | - If appropriate, what will the comparison group be? Does the comparison group have ‘real-world’ relevance? - Will the trial have “Reasonable treatment alternative intervention choices” (practical trial) - Or will be trial have “No-treatment or usual care comparison groups” (pragmatic trial) - Is there heterogeneity in the comparison group? How will this be documented or explored? (i.e., what is usual care?) |
| Recruitment of organisations to research involving web-based interventions | - In which geographical region(s) will recruitment take place? - How many organisations should be approached and/or recruited? Is a sample size calculation required? - What recruitment routes will be taken? Professional / business networks and bodies, local government, social media, print media, websites, charities. How will this be documented? - Who are the gatekeepers? Who provides consent on behalf of organisations? How will this information be recorded? - How will you recruit organisations? Who will approach them? - Who will determine whether organisations meet eligibility criteria? - Will recruitment of organisations be concurrent or rolling? - How and where will study information be communicated and how will consent be taken, and by whom? - Will non-responders be re-contacted? - How long will each organisation be in the study? - Will social marketing approaches be used to generate messages that appeal to groups that share specific characteristics? - How will anonymity (for organisations and employees) be highlighted? - How will potential discomfort with using technology be addressed to minimise technology-related barriers to take-up? - Can the benefits of web-based interventions be emphasised? - How will the researchers explain and detail the responsibilities of organisations and individuals (to help with informed decision-making)? - How will the researcher ensure credibility? (e.g., through relevant affiliation or endorsement). - How will the process of trials and randomisation be explained (i.e., to ensure perceptions of the ‘offer’ and ‘result’ are aligned). |
| Recruitment of employee participants to research involving web-based interventions | - How will end-users be reached? Directly or through employment settings? - How will you recruit participants? Is it opt-in or opt-out? Who will approach participants? - How many participants should be approached and/or recruited? Is a sample size calculation required? - Who will determine whether participants meet eligibility criteria? - Will recruitment of participants be concurrent or rolling? - How and where will study information be communicated and how will consent be taken, and by whom? - Will non-responders be re-contacted? - How long will each participant be in the study? - If study promotion occurs via employment settings, what marketing and messaging will occur? How will this be recorded? - How will recruitment of employees be managed? - Will end-users be representative of the population? - Is recruitment open, or limited to a known participant pool? Is it possible to calculate a response rate? Calculating the denominator can be challenging with open recruitment in real-world research. - What efforts will be made to address the digital divide? Differences in internet access, economics, and/or low computer literacy. - What efforts will be made to reach under-served employment settings and communities? |
| Outcomes and intended actions / behaviour change | - What participant outcomes will be measured, how, and over what timescale? - How will outcomes be measured at each time point? - What is positive change or action in this context? - Does the change or action impact others? How will the views or actions of others (towards the intervention and/or the participants’ resulting behaviours or actions) be documented or measured? |
| Attrition and retention | - What efforts will be made to minimise attrition and maximise retention? - What level of attrition is acceptable? This may relate to the intervention (e.g., accessing and engaging with the intervention), and research participation (e.g., completion of study outcome measures). - How will attrition be recorded? - Are there incentives or rewards for intervention participation and/or research participation/completion of outcome measures? Are incentives or rewards for individual participants, or host organisations? |
| User acceptance and satisfaction with intervention | - How is acceptance and satisfaction with the web-based intervention being measured? This relates to end-users, programme adopters (e.g., employers), healthcare providers (where relevant), policy makers. - Is the measurement or evaluation of acceptance informed by a theory or model? - Are there measures of technology adoption? |
| Routes to ‘real-world’ contexts and scale-up. | - How will routes to impact be explored? - Consider generalisability, implementation, cost-effectiveness, and social validity. |
